# Correction: A prospective, multi-site, cohort study to estimate incidence of infection and disease due to Lassa fever virus in West African countries (the *Enable* Lassa research programme)–Study protocol

**DOI:** 10.1371/journal.pone.0317720

**Published:** 2025-01-14

**Authors:** Suzanne Penfold, Ayola Akim Adegnika, Danny Asogun, Olufemi Ayodeji, Benedict N. Azuogu, William A. Fischer, Robert F. Garry, Donald Samuel Grant, Christian Happi, Magassouba N’Faly, Adebola Olayinka, Robert Samuels, Jefferson Sibley, David A. Wohl, Manfred Accrombessi, Ifedayo Adetifa, Giuditta Annibaldis, Anton Camacho, Chioma Dan-Nwafor, Akpénè Ruth Esperencia Deha, Jean DeMarco, Sophie Duraffour, Augustine Goba, Rebecca Grais, Stephan Günther, Énagnon Junior Juvénal Prince Honvou, Chikwe Ihekweazu, Christine Jacobsen, Lansana Kanneh, Mambu Momoh, Aminata Ndiaye, Robert Nsaibirni, Sylvanus Okogbenin, Chinwe Ochu, Ephraim Ogbaini, Énagnon Parsifal Marie Alexandre Logbo, John Demby Sandi, John S. Schieffelin, Thomas Verstraeten, Nathalie J. Vielle, Anges Yadouleton, Emmanuel Koffi Yovo, on behalf of the Enable Protocol authorship group

S1 and S2 Files are omitted from the list of Supporting Information. It can be viewed below.

## Supporting information

S1 File Data Management Plan. (PDF)

S2 File Statistical Analysis Plan. (PDF)
