# Supplementary figures and images for: A prospective, multi-site, cohort study to estimate incidence of infection and disease due to Lassa fever virus in West African countries (the Enable Lassa research programme)–Study protocol
Source: PLoS One. 2023 Mar 30;18(3):e0283643. doi: 10.1371/journal.pone.0283643 (PMC10062557; doi:10.1371/journal.pone.0283643)

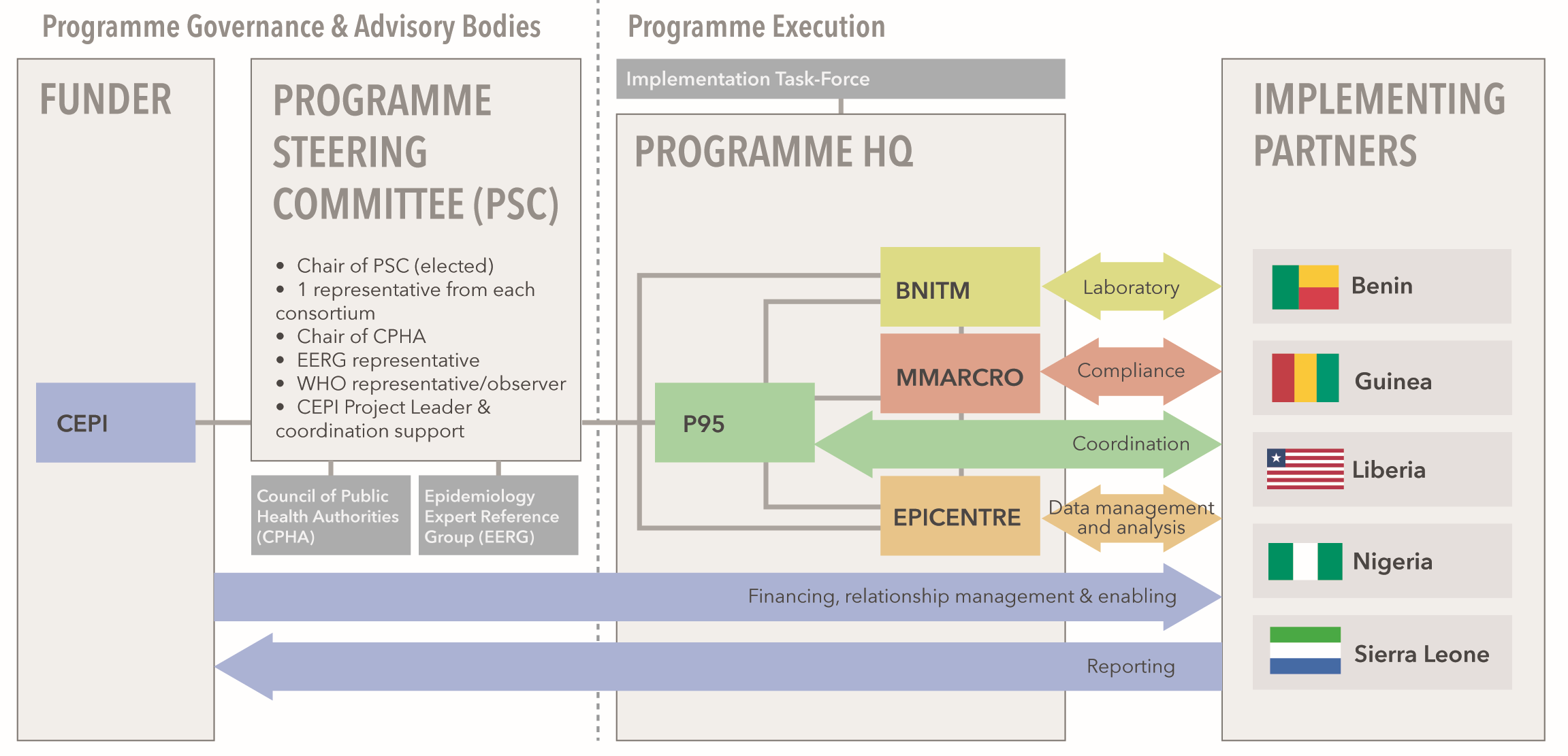

Supplement: S1 Fig — (TIFF) [file pone.0283643.s001.tiff]
