# Supplementary material for: A prospective, multi-site, cohort study to estimate incidence of infection and disease due to Lassa fever virus in West African countries (the Enable Lassa research programme)–Study protocol
Source: PLoS One. 2023 Mar 30;18(3):e0283643. doi: 10.1371/journal.pone.0283643 (PMC10062557; doi:10.1371/journal.pone.0283643)
Supplement: Attachment — Submitted filename: response to reviewers.docx [file pone.0283643.s002.docx]

Dr. Emily Chenette and Dr. George Vousden

*Editors-in-Chief*

*PLOS ONE*	         23^rd^ January, 2022

Dear Drs. Chenette and Vousden,

Thank you to both reviewers for reading our protocol papers. Please see our response to each of the points raised.

1. the study governance and roles of various partners - this part can be given in supplementary file

Response - This has been done

2. a map of the study sites can be given for better clarity

Response - This has been done

3. reason for including ">=2 years old" participants to be given

This was recommended by the local Lassa fever experts, who from their experiences, said that Lassa fever is very rare in the age group of less than two years. Also, community acceptability for blood draws from young children is low. Details on this rationale have been added to the inclusion/exclusion criteria on page 19 of the manuscript.

4. the inclusion criteria is fully not clear - are you going to recruit "healthy individuals" or LASV infection IgM/IgG positive individuals or LF disease patients (operational definition?)

Response - The text (page 19, line 5) has been amended to say ‘not febrile’ rather than healthy, which is more accurate and correct. This criterion was implemented to reduce the chance of recruiting active LF cases, but of course this would not exclude all with active infection as many are asymptomatic. It was not possible to conduct PCR tests on all participants at baseline.

5. This statement not clear: "draw the (smaller) LASV infection cohort as a nested cohort from the (larger, main) LF disease cohort" - shouldn't it be reverse as LASV infection is asymptomatic in approximately 80% of the cases. LF disease cohort should be nested in LASV infection cohort.

Response - Because the sample size required for the LASV infection cohort was smaller than that required for the LF disease cohort, nesting the disease cohort within the infection cohort was not possible. Lassa fever disease is the endpoint of future vaccine trials. Because it is unknown whether people can get Lassa fever disease more than once, baseline LASV seropositivity (IgG) did not preclude participation in the LF disease cohort.

6. Clustering effect how to adjust for in sample size not mentioned

Response – clustering considerations for the sample size calculation is mentioned in section ‘statistical analysis’, and is noted as a limitation as it was omitted: “The original sample size calculation in the protocol had several limitations: i) It could not account for the baseline LASV seroprevalence which is unknown in the study population. If the LASV seroprevalence in the study population is high, this is likely to reduce the number of people at risk in the cohort; ii) It did not take into account the clustering of participants within households and communities, which was necessary for feasibility and acceptance by the population; iii) It did not account for participant attrition during the 2-year follow-up period, which is likely to reduce the final cohort size for the analyses. As a result, the precision intervals specified in the protocol are likely to be narrower than those that will ultimately be obtained from the analyses after considering baseline seropositivity, clustering and attrition of participants.” As the other reviewer noted, “Although the justification for the sample size does not seem adequate, the figure seem(s) reasonably large for the desired precision.”

We have also addressed the style requirements of the manuscript, figures (also processed using PACE), file naming, and authorship group.

Yours sincerely,

Suzanne Penfold

P95 Epidemiology and Pharmacovigilance

Koning Leopold III laan 1

3001 Leuven

Belgium

email: suzanne.penfold@p-95.com
