# Supplementary material for: Correction: A prospective, multi-site, cohort study to estimate incidence of infection and disease due to Lassa fever virus in West African countries (the Enable Lassa research programme)–Study protocol
Source: PLoS One. 2025 Jan 14;20(1):e0317720. doi: 10.1371/journal.pone.0317720 (PMC11731728; doi:10.1371/journal.pone.0317720)
Supplement: S1 File — (PDF) [file pone.0317720.s001.pdf]

Data Management Plan

PROSPECTIVE MULTI-SITE COHORT STUDY TO ESTIMATE INCIDENCE OF INFECTION AND DISEASE DUE TO LASSA FEVER VIRUS IN WEST AFRICAN COUNTRIES

**Version Number and Date:**	Version 1.0, 31 May 2022
**Funding**	CEPI
**Study Principal Investigators:**	Prof Adebola Olayinka (NiLE Enable)<br>Prof Magassouba N'faly (GUILASSEPI)<br>Dr David Wohl (Liberia Enable)<br>Prof Akim Adegnika (LAVIHFiB)<br>Dr Donald Grant (Colect Enable)
**Data Management Contact(s):**	Robert Nsaibirni (Epicentre, Paris)<br>Robert.Nsaibirni@epicentre.msf.org

Summary of Changes:

Version	Version Date	Affected Section(s)	Summary of Revisions Made:
1.0	31 May 2022		**Initial Version**

APPROVAL SIGNATURES

Dr Robert Nsaibirni<br>Data Management Coordinator		Date	Jun 16, 2022
Dr Anton Camacho<br>Statistics Coordinator		Date	Jun 21, 2022
Prof Adebola Olayinka<br>PI Nigeria (NiLE Enable)	Bola Olayinka (Jun 23, 2022 16:44 GMT+1)	Date	Jun 23, 2022
Dr David Wohl<br>PI Liberia (Liberia Enable)	David Alain Wohl, MD (Jun 16, 2022 10:28 EDT)	Date	Jun 16, 2022
Prof Akim Adegnika<br>PI Benin (LAVIHFiB)	Ayola Akim ADEGNIKA (Jun 16, 2022 15:22 GMT+1)	Date	Jun 16, 2022
Dr Donald Grant<br>PI Sierra Leone (Colect Enable)	Donald S. Grant (Jul 6, 2022 06:22 GMT)	Date	Jul 6, 2022
Prof Magassouba N'faly<br>PI Guinea (GUILASSEPI)	Magassouba N'faly (Jul 5, 2022 16:00 GMT)	Date	Jul 5, 2022
Dr Suzanne Penfold<br>Study Coordinator (P95)	S. C. Penfold (Jun 20, 2022 11:12 GMT+2)	Date	Jun 20, 2022

TABLE OF CONTENTS

1 INTRODUCTION 5
2 STUDY SUMMARY 6
3 CLINICAL DATA MANAGEMENT SYSTEM (CDMS) 7
4 CASE REPORT FORMS 7
4.1 DEVELOPMENT AND APPROVAL 7
4.2 CRF FORMS AND STUDY VISITS 13
4.3 CRF AND CDMA CHANGE MANAGEMENT 15
4.4 COMPLETION 16
4.5 USER PRIVILEGES 18
4.6 CDMS LOGGING AND AUDIT TRAIL 21
4.7 CDMS SECURITY 22
5 DATA FLOW 22
6 DATA ENTRY AND DATA CLEANING 24
6.1 PRE-REQUISITES FOR SITE DATA ENTRY 24
6.2 CRF DATA ENTRY 24
6.3 EXTERNAL DATA – PREPARATION AND BULK UPLOAD 25
6.4 EXTERNAL DATA RECONCILIATION 25
6.5 COMPARING AND MERGING DATA 25
6.6 REAL-TIME DATA VALIDATION 25
6.7 MANAGEMENT OF QUALITATIVE DATA 25
7 DATA VALIDATION PROCESS 25
7.1 DATA QUERYING AND VALIDATION 26
7.2 DATA CLARIFICATION FORMS (DCF) 27
7.3 DICTIONARY AND CODING MANAGEMENT 27
8 DATA EXPORTS AND TRANSFER 27
9 DATA SAFETY MONITORING BOARD 28
10 DATA REVIEW 28
11 PROTOCOL DEVIATIONS 28
12 DATABASE CLOSURE 28
12.1 CLOSURE CHECKS 28
13 DATA STORAGE, ACCESS, AND ARCHIVING 29
13.1 STORAGE AND ACCESS OF ELECTRONIC DATA 29
13.2 STORAGE AND ACCESS OF CRFS 30
13.3 INTERIM/TEMPORARY DATA STORAGE 30
13.4 BACKUP AND RECOVERY OF DATA 30
13.5 ARCHIVING 32

ABBREVIATIONS AND DEFINITIONS

CDMA	Clinical Data Management Application
CDMS	Clinical Data Management System
CEPI	Coalition for Epidemic Preparedness Innovations
CDMA	Clinical Data Management Application
CDMS	Clinical Data Management System
CRF	Case report form (may be paper or electronic representation of the data collection tool)
Cross-check	An edit check that compares variables from different CRF pages or participants variables from different CRF pages to an algorithmic evaluation (term of convenience used to differentiate from multivariate edit checks)
DAG	Data Access Group
DCF	Data Clarification Form
DMP	Data Management Plan
DPO	Data Processing Officer
DQO	Data Quality Officer
DVP	Data Validation Plan
GDPR	General Data Protection Regulation
GCP	Good Clinical Practice
HIPAA	Health Insurance Portability and Accountability Act
LASV	Lassa Virus
LF	Lassa Fever
Multivariate Edit Check	An edit check (beyond a range check, valid value check, or required criterion) on a variable or set of variables on the same CRF page (term of convenience used to differentiate from cross-checks)
PHQ	Project Headquarters
PI	Principal Investigator
REDCap	Research Electronic Data Capture
SOP	Standard Operating Procedure

TMF	Trial Master File
WHO	World Health Organization

1 Introduction

Clinical research data management is the administration and supervision of tasks associated with the entry, transfer and/or preparation of source data and derived items for storage in a clinical trial database. It is an essential activity to ensure quality and accuracy of data collected during the study, the safety and confidentiality of participants and project compliance with good clinical practices (GCP).

This Data Management Plan (DMP) is designed as provided in Epicentre's institution wide standard operating procedures (SOPs) for data management. If for some reason(s) it becomes necessary to add information or processes that are not in conformity with the said SOPs, the reason(s) should be explicitly mentioned. This DMP will be updated until all data collection and data quality control tools are developed and validated, after which, any changes to these tools shall be reported in the data management report.

The DMP and its supporting documents describe the following essential project-specific data management procedures for the CEPI Lassa Epidemiology study:

- Case report form (CRF) development, validation, and completion guidelines.
- Data collection on paper and electronic CRFs
- Data entry and storage in a clinical data management application (CDMA) including:
  - System structure and development
  - User access roles
  - Data Dictionary
  - Database audit trail
  - Security, confidentiality, and backup
  - CDMA system validation
- Data flow, tracking, and reporting
- Data entry procedures, including:
  - Training plan of site data manager and field staff
  - Data entry guidelines
- Data validation procedures, including:
  - Data Validation Plan (DVP)

  - Real-time validation and data checks
  - Post-entry data cleaning and validation plan
  - Query management
  - Data review checks to support monitoring
- Data reporting, including:
  - Listings, charts and key progress indicators
  - Follow up
- Data coding, including:
  - Coding dictionary and versions and upgrade
- Electronic data transfer rules and reconciliation
- Database closure and locking
- Data storage and archiving

2 Study Summary

In order to assess the feasibility and prepare for potential future Lassa fever (LF) vaccine efficacy trials, it is necessary to gather epidemiological data on the background rates of infection and disease due to Lassa virus (LASV) in endemic areas, which are needed to assess the sample size requirements of Lassa vaccine efficacy trials. This is necessary because the incidence and spatial distribution of LF is likely to be significantly underestimated based on existing data, due to gaps in diagnostics, surveillance, and access to health services. The planned prospective multisite cohort study will provide incidence estimates of infection and disease due to LASV in multiple sites in west African countries to inform the design of future vaccine trials and Lassa vaccination strategy when suitable vaccines become available.

Conduct of the study will also help to strengthen site and investigator capacity to conduct vaccine trials, as well as to address several gaps identified in the World Health Organization (WHO) Lassa Fever Research and Development (R&D) Roadmap.

The core protocol[1] defines the key elements for a multi-country cohort study. Studies based on the same core protocol will be conducted in multiple sites across five countries in west Africa, which will enable LF

[11] CEPI LASSA EPI STUDY, v2.2_03Nov2020 Core Protocol

epidemiology to be assessed within each country and to be compared between sites and countries. The primary goal is to assess the feasibility of future vaccine efficacy trials, identify areas in which these trials might be optimally conducted and to inform the trial design.

3 Clinical Data Management System (CDMS)

Study data will be managed electronically using REDCap (Research Electronic Data Capture developed by Vanderbilt University), version v10.8.5, a clinical data management system hosted at Epicentre facilities in Paris.

REDCap is a secure, web-based application designed to support data capture for research studies, providing 1) an intuitive web and mobile interface for validated data entry; 2) audit trails for tracking data manipulation and export procedures; 3) automated export procedures for seamless data downloads to common statistical packages; 4) procedures for importing data from external sources; 5) offline data collection using the REDCap mobile app; and 6) controlled permissions defined user access.

REDCap is designed to comply with Health Insurance Portability and Accountability Act (HIPAA) regulations. REDCap by default is not compliant with Part 11 of title 21 of the Code of Federal Regulations, but we describe here and in the System Validation Plan and its supplements, the steps taken to achieve compliance.

At Epicentre, access to REDCap including the creation of user accounts with project creation and management privileges is centrally managed. However, in certain cases, the Clinical Data Management System (CDMS) once configured can be securely deployed in a decentralized architecture for use within a local area network without requiring access to the internet.

The project data manager(s) at Epicentre should have a user account with project creation and management privileges. Databases and data collection instruments for a project shall be created using either the account of the data manager or the data coordinator for the project.

4 Case Report Forms

4.1 Development and approval

The process of developing and validating of CRFs was adapted to the study and should be done in the following steps:

1. **Generic specification**: based on the latest version of the protocol (CEPI LASSA EPI STUDY, v2.0, Date 16-Nov-2018 Protocol), the different sources of data, and the data-categories[2] needed to respond to all study outcomes, including the timing and any special considerations that are required for their collection should be identified by the study Sponsor. The Sponsor (CEPI) should also identify the different user roles/groups that will need to handle study data from the filling in of eCRFs and/or paper forms through quality control, to data export, transmission, and data analysis. The data categories are identified in such a way as to ensure that not more data than those needed to respond to study outcomes shall be collected during the study.

2. **Country Adaptations**: The Sponsor (CEPI) should share the generic protocol and CRFs with the different countries' partners for adaptations. Clear instructions should be given as to the allowed set of changes to ensure that a pooled analysis is possible across all five countries.
3. **Final Specifications**: Working with the country teams and the Sponsor, the Epicentre DM team should ensure the database conform with the last and validated version of the paper CRF.
4. Once the country specific CRFs are obtained, the Epicentre Data Management team with input from the Sponsor and the Study Statistician will consolidate and re-organize the data elements (variables) in each data category and separate them into forms if not already done at the previous step. This logical separation as well as the ordering of questions should be based on (i) the protocol timing requirements (ii) the expected procedure for a participant's visits (iii) the time during the procedure when the data is sure to be available (iv) who will have to fill out the data into the CRF and (v) the ease of computerization and automated quality control checks and analysis.
   These data points are summarized in the "Functional Requirements" spreadsheet. The functional requirements document which also serves as the trial data dictionary should use the XML-based XLS-Form syntax. In this study, the functional requirements document will be generated automatically from REDCap. It will contain amongst other things and for each data point:
   i. the labels for the prompts and hints, and error/warning/info messages for the final CDMA
   ii. the variable type
   iii. the format and units
   iv. an indication of whether it is required

[2] Data described in general terms with minimal details, e.g., Demographic data, Medical Assessment, Vital Signs, etc.

v. all applicable skip logic

vi. all applicable validation logic

NB: All fields that cut across the different countries will as much as possible maintain the same variable name to ease review and statistical analyses from all the sites.

5. **CRF Design**: The tables below present the list of the tentative CRFs designed for each country. The final versioned list of CRFs will be maintained in a separate document.

Table 1. CRF Versions
**Required form for participants who complete each study visit. Other CRFs are supporting and are only to be used as needed for each participant.*

Nigeria

List of forms	
FORM 1: Enrolment and Baseline	FORM 10: Follow-up at 4 Months
FORM 2: Participant Contact Log	FORM 11: Study Termination
FORM 3: Follow up	FORM 12: Referral Form
FORM 4: Suspect Case Evaluation	FORM 14: Six Month Follow-up
FORM 5: Confirmed case Assessment	FORM 13a: Lab request form
FORM 6: Sample Collection	FORM 13b: Lab Test Results Serology
FORM 7: Confirmed case Treatment	FORM 13c: Lab Test Results RT-PCR
FORM 8: Data Extraction from admission record	FORM 13d: Lab Test Results NGS
FORM 9: Confirmed case Discharge	

Liberia

List of forms	
FORM 1a: Household Enrolment	FORM 11: Study Termination
FORM 1b: Participant Enrolment	FORM 12: Referral Form
FORM 2: Participant Contact Log	FORM 13a: Laboratory Request form
FORM 3a: CHV Two Week Follow Up Visit	FORM 13b: Lab Test Results Serology
FORM 3b: Fever Assessment	FORM 13c: Lab Test Results- Real Time RT-PCR and Malaria

FORM 4: Febrile Event Evaluation	FORM 13d: Lab Test Results Next Generation Sequencing
FORM 5: Confirmed Lassa Fever Case Report	FORM 13e: Lab Test Results- Blood Chemistry
FORM 6: Sample Collection	FORM 13f: Lab Test Results- Full Blood Count
FORM 7: Six Month Follow Up Visit	FORM 13g: Lab Test Results- Urinalysis
FORM 8: Lassa Hospital Data Extraction	FORM 13h: Lab Test Results- HIV and HBsAg
FORM 9: Lassa Fever Hospital Discharge	FORM 13i: Lab Aliquot Usage/ Destination
FORM 10: Lassa Fever 4 Month Post-Hospital Visit	

Sierra Leone

List of forms	
FORM 1a: Household Enrolment	FORM 9: Outcome
FORM 1b: Participant Enrolment	FORM 10: Four-month Follow-up
Form S0: Snakebite Burden	FORM 11: Study Termination
Form S1: Snakebite follow-up	FORM 12: Referral Form
FORM 2: Participant Contact Log	FORM 13a: Lab request form
FORM 3: Follow up	FORM 13b: Lab Test Results Serology
FORM 4: Suspect Case Evaluation	FORM 13c: Lab Test Results RT-PCR
FORM 5a: Confirmed case Assessment	FORM 13d: Lab Test Results NGS
FORM 6: Sample Collection	FORM 14: Risk Factors and SES assessment
FORM 7: Treatment	
FORM 8: Data Extraction	

Guinea

List of forms	
FORM 1a: Household Enrolment	FORM 12: Referral Form
FORM 1b: Participant Enrolment	FORM 13a: Lab request form
FORM 4: Suspect Case Evaluation	FORM 13b: Lab Test Results Serology

FORM 6: Sample Collection	FORM 13c: Lab Test Results RT-PCR
FORM 11: Study Termination	FORM 13d: Lab Test Results NGS
FORM 14: Six month Follow up Form	

Benin

List of forms	
FORM 1a: Household Enrolment	FORM 9: Hospital Discharge
FORM 1b: Participant Enrolment	FORM 10: Four-month Follow-up
FORM 2: Participant Contact Log	FORM 11: Study Termination
FORM 3: Follow up	FORM 12: Referral Form
FORM 4: Suspect Case Evaluation	FORM 13a: Lab request form
FORM 5: Confirmed case Assessment	FORM 13b: Lab Test Results Serology
FORM 6: Sample Collection	FORM 13c: Lab Test Results RT-PCR
FORM 7: Confirmed case Treatment	FORM 13d: Lab Test Results NGS
FORM 8: Data Extraction	

6. **CRF Approval**: The designed paper CRF forms should be shared for review to an enlarged group of members of the study team including representatives from CEPI, P95, MMARCRO, Epicentre, BNITM and study site staff (PI, Study coordinator and monitors). The reviewers should provide feedback, within 5 working days of receiving the CRF, by responding to the email (or by Sharepoint) with the forms containing their suggestions and comments in tracked changes. All comments and suggestions should be reviewed by the data management team and given a status, either integrated or reason(s) provided for why they were not integrated.
   At the end of this process and upon agreement of all parties, the final versions should be shared by official email by the sponsor to the study site. This email serves as the official approval of these initial CRFs.

7. **CDMA development and verification:** From within the study data manager's REDCap account, two separate environments should be set: one for the development and validation of the CDMA and the other for the production CDMA. The development will proceed as follows:

In development the data manager should:

1. Ensure that the approved study CRFs are created and populated with the corresponding sections and questions.
2. Ensure that as much as possible, skip-logic and validation constraints are programmed into the electronic forms.
3. Create data quality rules for relevance and validity of filled responses, as well as the coherence of the responses with respect to other questions in the same form.
4. Create the user groups with the permissions as described in Section 4.6 below.
5. Create the study visits described in Section 4.2 as REDCap “Study Events”.
6. Configure all data collection instruments for CRF forms that are used across several visits as repeating instruments.
7. Designate data collection instruments to the different study events.
8. Deactivate REDCap’s default reporting feature which includes information on the entire database.
9. Create any required custom reports for project follow-up and participant visit schedules.
10. Mark all fields with personal data as identifier fields

In the validations CDMA environment:

1. Copy the development project into a new project
2. Test all parts of the CDMA against the requirements and functional specifications of the CRFs. A separate validation plan, “Validation Plan” should be prepared for this purpose.

In the production CDMA environment:

1. Copy the validation project into a new project. The following items must be included in the copy:
   i. All data collection instruments
   ii. All users and user rights
   iii. All user roles

iv. All reports
v. All data quality rules
vi. All Project Folders

2. Create all custom data exports.

3. Move the project to production status.

4.2 CRF Forms and Study Visits

Table 2 below presents the list of visits, the CRF forms that should be available for each visit, and the number of times each visit is expected to reoccur.. The greyed-out form numbers in the third column are obligatory forms that must be completed during the corresponding visit while the others are forms that should be completed as needed.

Table 2: Visit schedules and available forms (cells in grey) for each visit. The "Visit Day Offset" indicates the number of days that separate a visit from a previous one. The "Offset Range" shows the acceptable shift for visit days.

Nigeria

Visit (Study Event)	Recurrence	Available CRF Forms (in grey)
Enrolment (HH and Members)	1	1, 6, 13a, 13b
Study Termination	1	11
Disease Cohort		
Active Follow Up	54	3
Passive Follow Up	n	2
Suspected Case Assessment	n	4, 6, 13a, 13b
Confirmed Case Management	1	5, 6, 7, 8, 9
Follow-up at 4 months post discharge	1	10
Infection Cohort		
Infection Cohort follow-up (T6, T12, T18, T24)	4	6, 13a, 13c, 13d, 14

Liberia

Visit (Study Event)	Recurrence	Available CRF Forms
Enrolment (HH and Members)	1	1a, 1b, 6, 13a, 13b

Study Termination	1	11
Disease Cohort		
Active Follow Up	54	3a, 3b
Passive Follow Up	n	2, 3b
Suspected Case Assessment	n	4, 6, 13a, 13b
Confirmed Case Management	1	5, 6, 8, 9
Follow-up at 4 months post discharge	1	6, 10
Infection Cohort		
Infection Cohort follow-up (T6, T12, T18, T24)	4	6, 7, 13a, 13c, 13d, 13e, 13f, 13g, 13h, 13i

Sierra Leone

Visit (Study Event)	Recurrence	Available CRF Forms
Enrolment (HH and Members)	1	1a, 1b, 6, 13a, 13b, 14, S0
Study Termination	1	11
Disease Cohort		
Active Follow Up	54	3
Passive Follow Up	n	2, 3
Suspected Case Assessment	n	4, 6, 13a, 13b
Confirmed Case Management	1	5a, 7, 8, 9
Follow-up at 4 months post discharge	1	10
Infection Cohort		
Infection Cohort follow-up (T6, T12, T18, T24)	4	15, 6, 13a, 13c, 13d
Snake bite 12 Months Follow up		
Follow up each 12 month		S1, 14

Benin

Visit (Study Event)	Recurrence	Available CRF Forms
Enrolment (HH and Members)	1	1a, 1b, 6, 13a, 13b
Study Termination	1	11

Disease Cohort		
Active Follow Up	54	3
Passive Follow Up	n	2 | 3
Suspected Case Assessment	n	4 | 6 | 13a | 13c
Confirmed Case Management	1	5 | 7 | 8 | 9
Follow-up at 4 months post discharge	1	10
Infection Cohort		
Infection Cohort follow-up (T6, T12, T18, T24)	4	6 | 13a | 13b | 13d | 14

Guinea

Visit (Study Event)	Recurrence	Available CRF Forms
Enrolment (HH and Members)	1	1a | 1b | 6 | 13a | 13b
Study Termination	1	11
Infection Cohort		
Infection Cohort follow-up (T6, T12, T18, T24)	4	4 | 6 | 13a | 13c | 13d | 14

4.3 Study Dashboards Study Dashboards

Two dashboards will be developed by Epicentre for each site: a reporting dashboard and a QC/Scheduling dashboard. The reporting dashboard will contain key performance indicators on the study progress in the different sites. The quality control (QC) and scheduling dashboard will provide an interactive medium to share automated queries and participant visit schedules with the study sites.

The scheduling dashboard will provide the follow-up schedules for each participant and for each study day. It equally indicated the follow-up window precising the open date and the close date. Colour codes are used to indicate follow-up priorities with respect to closing of follow-up window: Green (4-3 days left) Yellow (2days left) Red (1-0 day left).

4.4 CRF and CDMA change management

If it becomes necessary to make changes to the CRF and CDMA after data collection has started, the following procedure should be followed:

1. A change request is initiated using the “CRF/CDMA change request and implementation” form.

2. The CRF change group will evaluate the pertinence of the change, and carry out a risk assessment, then indicate in the appropriate section of the form whether or not the change is approved.
3. If the change is approved, it is made to the paper CRF then the development CDMA. After review and approval by P95, Epicentre and study staff, the final version and tracked changes versions of the CRFs are both stored within the trial master file (TMF) or study OneDrive and the change tracker file updated.
4. The functional specifications are updated as necessary, and a new version created.
5. The changes are validated, and the validation documented in a similar way as the initial validation of the CDMA.
6. The change request and completion form are completed and signed by the designated signatories of the “approval of case record form(s) design” document, to be filed in the TMF.
7. Once the changes are satisfactorily validated, the updated data dictionary is uploaded into the production CDMA or the exact changes are replicated directly in the production CDMA. Whatever method is used, at the end, the online instruments and the data dictionary should always match.
8. All old paper CRFs are withdrawn from the study site by the site data manager and the new ones provided.
9. To carry out CDMA versioning, the REDCap Project metadata (dictionary, forms, roles, data access groups, etc.), the project data and the audit logs are backed up prior to pushing the changes to production and immediately after the changes are pushed to production. These backups are versioned, dated, encrypted, and stored securely in Epicentre’s backup servers.

4.5 Completion

Data will be directly captured electronically into REDCap. While form 13a will be filled on paper and transcribed later. Form 13a will be partially filled by the field worker and completed and at the laboratory. In case of issues with the tablets, the paper CRF will be used as backup to collect data for subsequent entry into REDCap.

For performance and usability purposes, where possible, participant records shall be distributed across tablets. This means that each tablet will only contain a subset of the participants and depending on its intended use (field, laboratory, health facility), will only contain the relevant forms. The Epicentre data management team will work with the site teams to define a distribution framework that fits with their team organisation and how different study visits and procedures are performed.

Table 2 below summarises presents the users who will be authorised to fill different study CRFs. Study participants will not complete any CRF forms themselves.

Table 2. Study roles authorized for CRF completion.

CRF Form Name	Authorized Users	Countries
FORM 1: Enrolment and Baseline	Field Workers	N
FORM 1a: Baseline Household Enrolment	Field Workers	ALL
FORM 1b: Baseline Participant Enrolment	Field Workers	ALL
FORM 2: Participant Contact Log (Passive FU)	Field Workers	N, L, B, SL
FORM 3: Active Follow Up	Field Workers	N, B, SL
FORM 3a: Two Week Follow Up	Field Workers	L
FORM 3b: Fever Assessment	Field Workers	L
FORM 4: Febrile Case Evaluation	Field Workers/Facility staff/Field Workers	ALL
FORM 5: Confirmed Lassa Case Report	Field Workers/Facility staff/Lab Staff	N, L, B, SL
FORM 6: Sample Collection	Field Workers/ Phlebotomist	ALL
FORM 7: Confirmed Case Treatment Form		N, B, SL
FORM 7: Six Months Follow Up Form	Field Workers/Health Facility staff	L
FORM 8: Lassa Hospital Data Extraction Form	Field Workers/ Health Facility staff	N, L, B, SL
FORM 9: Lassa Hospital Discharge	Field Workers/ Health Facility staff	N, L, B, SL
FORM 10: Lassa Case – FU AT 4 MONTHS	Field Workers/ Health Facility staff	N, L, B, SL
FORM 11: Study Termination	Field Workers/ Health Facility staff	ALL
FORM 12: Referral Form	Field Workers	N, L, B, SL
FORM 13a: Lab request form	Field Workers/ Phlebotomist/Lab staff	ALL
FORM 13b: Lab Test Results Serology	Lab Staff	ALL
FORM 13c: Lab Test Results RT-PCR	Lab Staff	ALL
FORM 13d: Lab Test Results NGS	Lab Staff	ALL
FORM 13e: Lab Test Results- Blood Chemistry	Lab Staff	L
FORM 13f: Lab Test Results- Full Blood Count	Lab Staff	L
FORM 13g: Lab Test Results- Urinalysis	Lab Staff	L
FORM 13h: Lab Test Results- HIV and HBsAg	Lab Staff	L
FORM 13i: Lab Aliquot Usage/ Destination	Lab Staff	L
FORM 14*: Six month Follow up Form	Field Workers	N, B, SL, G

Key:
N: Nigeria; **L**: Liberia; **B**: Benin; **SL**: Sierra Leone**; G**: Guinea

The "CRF Completion guideline" will be developed jointly by the data manager, the local Sponsor and the program implementation partners to ensure accurate and consistent completion of each CRF form according to the Epicentre "SOP *EPI-SOP-DM05-Data entry and processing"*. Training should be provided

to the study staff, investigators, nurses, phlebotomist, and lab technician prior to enrolment. There must be evidence of the training on the completion of the CRF i.e. an agenda, signed list of participants trained and copies of material used for the training.

All CRF forms are equipped with a tracking section at the end. All persons completing the CRF forms will indicate the initials of their names and date of completion before submitting the completed forms.

4.6 User privileges

User privileges help to ensure that REDCap users only have access to data, information, and features that they need within the application. In this study, each site PI validates and signs a Data Access Matrix prepared by Epicentre and an Access Grant Revoke form which specifies the access rights for each role and the site staff members assigned to each role. This access right matrix once signed is then used by Epicentre to create access roles and permissions to the REDCap and the study dashboards. To access the study REDCap CDMS and the study dashboards, all users must have a valid username and password, provided by the Epicentre System Administrator. Tables 4a, 4b, and 4c below are a snapshot of the initial Data Access Matrix describing user roles and permissions as adopted for this project. Each authorized user will be assigned to one of these pre-defined roles. Country specific Data Access Matrices will be created and maintained as part of the country specific documentation and further changes will only be captured in the latter.

The following good practices for password management and protection of individual workstations will be required for all staff members:

1. Passwords must be complex and not easily guessed
2. Passwords should never be stored in browsers,
3. Always lock screen when leaving workstation,
4. Never share password with a colleague or any other person for any reason whatsoever,
5. Do not write passwords down and keep around the workstation,
6. It is recommended to change passwords every three months.

Table 4a: Project Roles and Permissions for REDCap

**Task/Role**	**PI & Study Coord. (1, 4)**	**Field Worker (5)**	**Health Worker (12)**	**Laboratory (7, 8, 9)**	**Site DM (10)**	**Epicentre DM (15)**	**Epicentre DM Coord.**	**Monitors (13, 14)**	**Statistician (17)**	**Programmer (18)**
A. Access Control										
Create/Remove Users							X			
Grant/Revoke access						X				
(Re)Assign users to groups (see Section 4)					X	X				
B. Project Development										
Project design and setup						X				
Logging/Audit trails					X	X				
CRF change implementation					X	X				
C. Data Collection and Records Management										
Create Records (participant or sample)		X								
Collect data for a record		X	X	X						
Update Record		X		X	X					
View record (read-only)	X							X	X	X
Complete paper forms			X	X						
Rename record					X	X				
Delete record						X				
Change record's DAG					X	X				
Lock/unlock records					X	X				
D. Data Export and Reporting										
Create exports and reports	X				X	X				
Export data (de-identified only)	X				X	X			X	X
E. Quality Control										
Raise queries								X		
Resolve queries		X	X	X	X					

Table 4b: Roles and Permissions for the study dashboards

**Institution / Country**	**Role(s)**	**Dashboards**			**Sites Accessible via Dashboard**						
		**Queries**	**Scheduling**	**Reporting**	**EDO**	**OND**	**EBO**	**BEN**	**SLE**	**GUI**	**LBR**
CEPI	PHQ Oversight			X	X	X	X	X	X	X	X

Institution / Country	Role(s)	Dashboards			Sites Accessible via Dashboard						
		Queries	Scheduling	Reporting	EDO	OND	EBO	BEN	SLE	GUI	LBR
P-95	PHQ Oversight, Study Monitor	X	X	X	X	X	X	X	X	X	X
BNITM	PHQ Oversight			X	X	X	X	X	X	X	X
Epicentre	PHQ Oversight			X	X	X	X	X	X	X	X
Epicentre	DM and Statistics	X	X	X	X	X	X	X	X	X	X
Mmarcro	Study Monitor	X		X	X	X	X	X	X	X	X
Mmarcro	CRA Nigeria	X		X	X	X	X				
Mmarcro	CRA Benin	X		X				X			
Mmarcro	CRA Sierra Leone	X		X					X		
Mmarcro	CRA Guinea	X		X						X	
Mmarcro	CRA Liberia	X		X							X
Nigeria	NCDC Study Coordinator	X		X	X	X	X				
Nigeria	Site DM NCDC	X	X	X	X	X	X				
Nigeria	Site DM EDO, Site Monitor	X	X	X	X						
Nigeria	Site DM OND, Site Monitor	X	X	X		X					
Nigeria	Site DM EBO, Site Monitor	X	X	X			X				
Nigeria	EDO PI, Study Coordinator, Lab	X		X	X						
Nigeria	OND PI, Study Coordinator, Lab	X		X		X					
Nigeria	EBO PI, Study Coordinator, Lab	X		X			X				
Benin	PI, Study Coordinator, Lab	X		X				X			
Benin	Site DM, Site Monitor	X	X	X				X			
Sierra Leone	PI, Study Coordinator, Lab	X		X					X		
Sierra Leone	Site DM, Site Monitor	X	X	X					X		
Guinea	PI, Study Coordinator, Lab	X		X						X	
Guinea	Site DM, Site Monitor	X	X	X						X	
Liberia	PI, Study Coordinator, Lab	X		X							X
Liberia	Site DM, Site Monitor	X	X	X							X

Some of the roles mentioned above might have different nomenclature in the different countries. The table below defines roles as used in this study.

Table 4c: Project Roles

Role	Definition
PI	Site principal investigator
Project manager	Representative of the study site who is responsible to the organisation of the work at the site, hiring personnel, organisation of procurement and execution of the project against the deadlines
Study coordinator	Representative of the study site who is responsible for the implementation of the study, organisation of the work, implementation of study procedures and fulfilment of reporting requirements to the study HQ
Field supervisor	Employee of the study site who is responsible for the training of field workers, organisation of their work and supervision
Field worker	Employee of the study site who is responsible for the enrolment of participants and their follow-up, as well as meeting all reporting requirements.
Health worker	Employee of the healthcare facility to which study participants can be referred for diagnosis and treatment
Phlebotomist	Employee of the study site who is responsible for collection, handing and storage of biological specimens
Laboratory supervisor	Employee of the laboratory processing specimens from study participants, responsible for organisation of the laboratory, training of laboratory technicians, safety and reporting requirements fulfilment
Laboratory technician	Employee of the laboratory processing specimens from study participants, responsible for performing tests and reporting, and entering data into REDCap.
Site Data manager	Employee of the study site who is responsible for the implementation and maintenance of the data management system, backup of data and quality assurance of collected data
Epicentre DM	Epicentre staff responsible for the design of paper CRF, the development and validation of eCRFs and the overall management of the project data.
Epicentre DM Coord.	Epicentre staff responsible for the overall maintenance of REDCap and the hosted databases include backup and recovery in case of disaster.
Statistician	Epicentre staff responsible for the development of the statistical analysis plan and the review and analysis of collected data.
Programmer	Epicentre staff responsible for programming automated reporting and QC tools for the study.

4.7 CDMS Logging and Audit Trail

REDCap maintains a built-in audit trail that logs all user activities and includes contextual information (the project, record ID, event name, and date/time of access) the username, the type of action performed

(page views, instrument design, record creation, update, deletion and import and export activities), and, if update or deletion of records, the list of changes made with the appropriate field comments.

The audit trail can be viewed within a project in REDCap or downloaded as a CVS file by users with adequate privileges (see country specific Data Access Matrix).

4.8 CDMS security

MSF/Epicentre, Paris has a license to use REDCap and is therefore responsible for providing the proper IT infrastructure and environment to host the application. This includes the webserver and database server, as well as the connection between the two and the connection of the webserver to the end-user. The REDCap application is written in PHP and delivered by the NGINX web server. The underlying database server is MySQL. The web server exists in the DMZ and all data transmitted between the client browser and web servers are encrypted using an SSL connection. The servers are protected by both a hardware security system and a web application security system, with network security audits and updates and daily data back-ups stored securely off-site.

As described above, only authorized people will have access to the data through REDCap's online or mobile-app interface. The data management team's computers at each study site are password protected and the local network through which the browser is accessed is protected by firewalls.

5 Data Flow

The following flowchart describes how data flows in the context of the study from filling-in the CRFs through storage and quality control to reporting. More detailed descriptions of visit specific data flows can be found in the SOP "EPI-SOP-A.01d CRF completion v1.0 27Nov20".

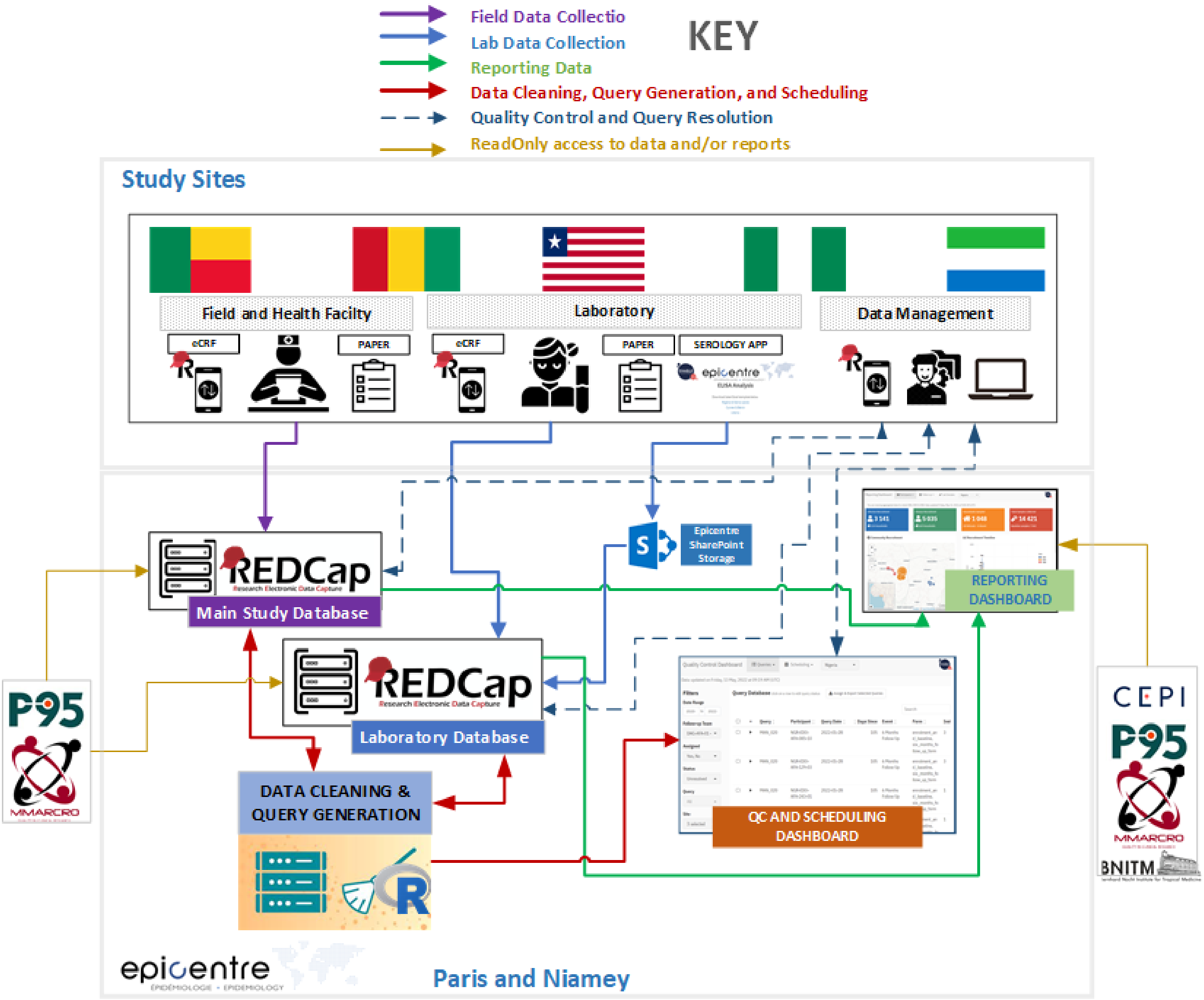

Figure 1: Data Flow Chart

1. Epicentre stores all study data within its secured cloud infrastructure located and Paris.
2. Measures are taken to store and process data in accordance with good clinical data management practices (GCDMP) and the general data protection regulations (GDPR).
3. Each country will have a separate set of study CDMAs (Main database and Laboratory database).
4. All data is transmitted via encrypted HTTPS connections.
5. Countries (site staff with appropriate permissions) will be able to access the CDMA and export data (PDF, Excel, Stata, ISPS) at any time, but, once exported Epicentre has no liability or control over that data, how it is processed, and where it is stored.

6. Theft protection and tracking, and app-use control software will be installed (FindMyDevice, AppsLock,…) in all mobile devices used for data collection.
7. SOPs and trainings will be used to (re)enforce human/infrastructure elements of security.

6 Data Entry and Data Cleaning

6.1 Pre-requisites for Site Data Entry

The site users (the Data Manager and the data entry clerks) must be trained in REDCap prior to being granted permission to work in the production version of the CDMA. Staff trainings will also include an overview of the following documents: this data management plan, the study protocol, the data validation plan, Data Entry Guidelines, study SOPs, and the General Data Protection Regulations. Evidence of the training including the agenda, the signed list of participants trained and copies of material used for the training will be retained within study documentation.

6.2 CRF Data Entry

Data is primarily entered in real time, by field workers and healthcare workers directly into the electronic CRFs on tablets and sent to the server. Data from paper forms will be entered by trained data entry clerks only on tablets which is sent to the server daily. Data Entry Guidelines will be developed and provided to all study staff involved with data entry, following Epicentre's institution wide SOP for Data Entry (*EPI-SOP-DM05-Data entry and processing*). Data will be directly captured into the CDMA during the interview with the participant.

Data entry processes will be supervised by the site Data Manager or delegate (data entry supervisor). Entry will be completed at the end of the interview and sent on the server while leaving the household or at the end of the working day. The fieldworker/lab staff/health worker should check at the end of each interview that at least all the mandatory fields are filled and save the form as 'Unverified'. The site data manager will later check data consistency and move the form status to 'Complete'. Each fieldworker/lab staff/health worker is assigned to one Data Access Group and cannot view entries of other entrants in the other teams.

Entry clerks will also be able to create new records and modify existing records that they entered but will not be able to delete records or modify records entered by other clerks.

6.3 External data – preparation and bulk upload

Serology tests will be processed using a web application designed specifically for this purpose and the generated results will be automatically imported into REDCap. This process is described in the "EPI-SOP-B.16 Entry and Processing of Serology Results" standard operating procedure.

6.4 External data reconciliation

NA

6.5 Comparing and Merging Data

NA

6.6 Real-time data validation

Quality control will be performed throughout the study on all completed CRF Forms. See Data Validation section below for more information on data queries.

Many of the real-time data validation rules are in place to identify simple entry errors (e.g. if the data entry clerk enters an incorrect participant ID). Any field that must be entered in the CRF will be marked as "Required" in the REDCap database, and an alert will appear if the field is left blank. Furthermore, branching logic ensures that only the appropriate fields appear for data entry, based on previous responses recorded for the participant so that only all appropriate data are recorded for each participant. REDCap will also generate alerts if a value falls outside the expected range, is entered in an incorrect format, or violates any one of the user-defined validation rules, which primarily test for concordance across different CRFs.

The data entry clerk will be able to modify any invalid response at the time of entry (e.g., enter data from a field erroneously left empty during initial entry. If REDCap flags an alert which cannot be resolved by the DPO/DQO (e.g., the information entered on the CRF violates a cross-check rule), the data entry clerk should liaise with the data manager who will make the update.

6.7 Management of Qualitative data

N.A.

7 Data Validation Process

Edit checks, skip constraints, coherence checks and required question constraints are explicitly implemented in the CDMA and are checked in real-time during data entry following the procedures described in the SOP "*EPI-SOP-DM02-Validation of CDMAs*." As described above, there are several real-time data validations in place. During entry, different alerts will appear in cases of invalid formats, missing values that should be entered during the visit (i.e., fields marked as "Required" that are not entered), invalid ranges, and multivariate cross-checks within and across CRFs in the same record.

7.1 Data Querying and Validation

Post-entry data cleaning will be performed on a regular basis. All data will be automatically extracted from the CDMA system into the data quality dashboard, where Quality checks will be performed as per Data Validation Rules. For each country, a list of queries (missing fields, fields inconsistency, …) will be confined in the 'data validation rules' files which will be used and executed to sort out all the queries and inconsistencies in the database. At this stage, any inconsistencies that were missed during data entry will be identified and resolved. The code to identify these inconsistencies will be developed in advance of any data cleaning activities; this will ensure consistency in data cleaning efforts and allow for a thorough consideration of potential errors and inconsistencies that need to be checked. Any changes made to the data cleaning scripts will be documented and applied retroactively to previously cleaned data. Once the DM logs into the quality control dashboard, s/he will be able to see the list of queries identified by the query ID and other information related (Participant, Query date, event,). The DM will assign each query to the data entry clerk, export them, and give them to each fieldworker/lab staff/health worker. They will then check the information in REDCap mobile App, and if any update is necessary, this should be done and then synchronized so that the information is considered on the server. Sometimes, queries might not be resolved by the field worker (like participant temperature), in this case, the Site Data Manager must mark it as resolved and this will be considered as true missing data.

Data quality rules will be developed and implemented into a query dashboard to check missing data, data inconsistencies and discrepancies in the data base. These rules/scripts will be executed or run every hour to highlight data issues (eg missing, incorrect format, inconsistencies…). Each query has an ID, followed by detailed information (Participant ID, date, event, form, instance) which eases the identification of the query and the participant. Sites DMs will be able to assign queries to the concerned user for resolution. For a better understanding, a description of the query is provided with a suggestion for resolution. Queries can be grouped by many parameters like user ID, query status, Site, date range, etc and exported in many

formats (html, xls). When data is corrected on the database, the Query dashboard updates automatically to indicate that the query has been resolved. A query metrics is provided to give key indicators on the evolution of query resolution from the start of the study and can be disaggregated by database, site, and community.

7.2 Data clarification forms (DCF)

NA

7.3 Dictionary and coding management

NA

8 Data exports and transfer

Data exports, storage and transfer will be done following the procedure described in the institution wide SOP "EPI-SOP-A.01h Guideline for sending data". In addition, data exports will only be possible using the pre-configured custom exports and only by authorized individuals or roles. All study staff members with export capabilities or with whom exported data can be shared will have to sign the "Acknowledgement of retention of project data" document, that contains the type of data they will have access to, the purpose for the access, a statement assuring that they will only use the data for the said purposes and will only hold the data for the period necessary to achieve these purposes.

Through a reporting dashboard, several reports will be designed in advance of data collection, and regularly used in the completion of study activities (e.g., a report generating the recruitment timeline per site, the achievement in terms of recruitment per site and community). These reports will be designed by the Data Manager and the Sponsor team and generated as needed by users with access to the report.

Requests from unauthorized staff members (e.g. Collaborator, Research Nurse), to access reports or exports will be made to the Sponsor team and Epicentre Data Manager Coordinator. The requestor must provide detailed information as to what specific data they need, why they want this data, and how it will be stored. Such requests can only concern de-identified data and will be validated by the PI and executed and logged by the data manager.

Data can be shared with implementing partners for operational use (e.g. for monitoring visit) via a secured FTP (File Transfer Protocol) server. Datasets accessed through FTP servers will be pseudonymized, encrypted at rest and in transit and a log of all actions will be maintained.

Countries will have access and be able to securely export the entire study data via the CDMA in either pseudonymized or raw format (so no ftp required for countries). The country specific Data Access Matrix indicates which roles have these export permissions.

9 Data safety monitoring board

NA.

10 Data review

Overall data reviews will be done by Epicentre regularly to evaluate the correctness of data collected, tendencies and give recommendations to the sites. This will be besides the real time continuous data quality checks. These reviews will be presented to the study PHQ as well as to the implementing Partners. A reporting dashboard will provide an overall view of project status by giving real-time indicators as data is being entered synchronised from the field for example number enrolled, follow-up visits, suspect cases, number of samples collected, refusals, maps, etc. All implementing partners should by default access data through dashboard and any additional feature needed by an implementing partner will be analysed and may be added.

11 Protocol deviations

During the process of data cleaning and more specifically the query generation, if a query is flagging a protocol deviation, it will be listed and be shared with the monitor of the study (MMARCRO Clinical Research Associates) for reporting to sponsor and regulatory authorities.

Before the database lock, all deviations will be reviewed with the monitor and the study team, and the final list of deviations (minors and majors) will be shared with the study statistician to be considered in the per protocol population definition. Deviations will not be entered in the database.

12 Database Closure

12.1 Closure Checks

In addition to the data validation schemes described above and as per the institution wide SOP, "*EPI-SOP-DM07-Preparing data for analysis*", the following checks will be performed before closure of the database:

- Check that all expected CRFs have been provided, monitored and entered.
- Confirm that all REDCap records are marked as “Complete” and locked.
- Check that the queries have been resolved.
- Check for value formatting problems in database exports.

The database will be locked following consultation with CEPI, and the organization of a Data Review Meeting chaired by the Epicentre Data Manager and Statistician and the approval of a Database Lock form. The data coordinator will be responsible for REDCap database lock. First, the data coordinator will remove the right to modify user rights from all other users. The data coordinator will then (1) remove the right to create, modify, or delete records; (2) remove the right to import data; (3) change the data entry right to “Read Only” for all users. The data coordinator will document that each authorized user no longer has user rights that allow for data entry or modification, thereby locking the database to future changes.

Once the database lock has occurred, any necessary changes to the data must be done through a properly approved and documented unlocking and data amendment process as described in the “SOP DM07: Preparing Data For Analysis.” A "Request to amend data after database lock" form is used to log and track these changes. This form contains the exact change, the reason for the change, the person requesting and the person validating the change, how the change will be implemented and a section to indicate when the change is implemented. Once the changes are made, the database is relocked following the same process as with the initial lock.

13 Data storage, access, and archiving

13.1 Storage and access of electronic data

Electronic data will be stored on the database server and is only accessible by authenticated users through a secured network, as described above. Physical access to the database server is restricted only to the IT Manager and Epicentre Data Manager Coordinator. Data is backed up daily to a secure, off-site location. At the end of the study, the data will be transferred in electronic format to the Sponsor and then archived following procedures in place at Epicentre.

13.2 Storage and access of CRFs

If for any reason, the paper CRF is used (a REDCap bug, tablet disability…), data should be captured into REDCap once the issue is addressed and the printed paper CRFs used, will be stored in a locked records room. This room is only accessible to designated study staff. Site specific procedures should be put in place to describe the records room and the storage, handling, and processing of study documents.

Each study participant will have a unique file numbered according to the primary participant identifier (participant ID). These files can be easily retrieved from the records room whenever they are needed by the authorized persons. Study staff shall always record in the logs whenever a file is taken out or brought back to the records room to ensure proper tracking of CRFs. After the completion of the study and locking of the study database, all the study specific materials (such as printed CRFs, etc.) will be sent to CEPI for archiving or will be destroyed upon authorization by CEPI. These records are kept in the archive room in following GCP guidelines.

13.3 Interim/Temporary data storage

As per the institution wide SOP "*EPI-SOP-DM06-Receiving and Uploading Data*", a secured storage space shall be created for read-only retention of exported project data. This storage place will have limited access, encrypted data-at-rest, encrypted transfers and provide detailed audit trails.

As described in Table 3 above, only the data managers and the study statistician will have data export rights in the project CDMA. Data can be exported for data validation/cleaning:

- Execution of post-entry quality control scripts
- Manual backup of project data

Data can also be exported for the purposes of analysis: interim or ad-hoc analysis, and final data analysis. Any project staff assigned to Data Manager or Statistician roles must complete and sign the "Attestation of Retention of Project Data" form along with the Confidentiality form for the project and the research center which must be completed and signed by all project staff members.

13.4 Backup and Recovery of Data

As per the institution wide SOP "*EPI-SOP-IT04-Backup and recovery of systems*", data should be backed-up such that not more than half a day's work is lost in the event of an incident.

1. **Backup**

Study data is backed up using Epicentre's backup procedures as follows:

a. The Entire REDCap Database is backed up regularly. The procedure is as follows:
   i. A full backup of the entire REDCap system database is done every day at 00h00 GMT.
   ii. Incremental backups are done hourly following every full backup till the next full backup.
   iii. At the end of the day, the full backup with its incremental backups are placed in a single dated folder, compressed, and encrypted.
   iv. This daily encrypted backup is securely transferred to Epicentre's backup server. Only backups for the last week are kept in the project server.

b. The Project Database is backed up as follows:
   i. Each day, a full back up of the study CDMA is created every hour. This backup contains a CDISC compatible XML file of the entire REDCap project instance including: forms, data and metadata; the audit trail and the list of forms.
   ii. At the end of the day, the backup files are placed in a dated folder, compressed and encrypted.
   iii. This daily encrypted backup is securely transferred to Epicentre's backup server. Only backups for the last week are kept in the project server.

c. All backups are kept for at least three (3) months.

2. Recovery

- If the entire REDCap Database fails, obtain the most recent "Entire REDCap Database" backup file, decrypt it and follow recovery procedures to reinstall the database.
- If only the project database gets corrupted or needs to be rolled back for some reason:
- use the most recent "Project Database" backup file, or any other backup as necessary,

- decrypt the file to obtain its contents (project XML file).
- from REDCap:
  - delete the current project from REDCap entirely,
  - create a new project using the project XML file,
  - REDCap creates a project exact to the time the recovered backup file was created.

- Recovery tests are performed and documented quarterly by the Data Management Systems Administrator.

13.5 Archiving

The final locked study database will be archived at Epicentre and transmitted via a secured means to the Local Sponsors (Study Sites) for final archiving. Archiving will be done following procedures in force in the corresponding institutions and/or countries.

DMP Lassa - signatures

Final Audit Report 2022-07-06

Created:	2022-06-16
By:	Marine Durthaler (marine.durthaler@epicentre.msf.org)
Status:	Signed
Transaction ID:	CBJCHBCAABAALSikNrqYw7TQBIOMJpdH8Vn600kYYQZF

"DMP Lassa - signatures" History

Document created by Marine Durthaler (marine.durthaler@epicentre.msf.org)
2022-06-16 - 2:14:48 PM GMT- IP address: 84.14.94.68

Document emailed to Robert NSAIBIRNI (robert.nsaibirni@epicentre.msf.org) for signature
2022-06-16 - 2:17:39 PM GMT

Document emailed to Anton CAMACHO (anton.camacho@epicentre.msf.org) for signature
2022-06-16 - 2:17:39 PM GMT

Document emailed to adebola.olayinka@ncdc.gov.ng for signature
2022-06-16 - 2:17:40 PM GMT

Document emailed to david_wohl@med.unc.edu for signature
2022-06-16 - 2:17:40 PM GMT

Document emailed to ayola-akim.adegnika@uni-tuebingen.de for signature
2022-06-16 - 2:17:40 PM GMT

Document emailed to donkumfel@gmail.com for signature
2022-06-16 - 2:17:40 PM GMT

Document emailed to cmagassouba01@gmail.com for signature
2022-06-16 - 2:17:40 PM GMT

Document emailed to suzanne.penfold@p-95.com for signature
2022-06-16 - 2:17:40 PM GMT

Email viewed by ayola-akim.adegnika@uni-tuebingen.de
2022-06-16 - 2:18:37 PM GMT- IP address: 66.249.93.174

Document e-signed by Ayola Akim ADEGNIKA (ayola-akim.adegnika@uni-tuebingen.de)
Signature Date: 2022-06-16 - 2:22:58 PM GMT - Time Source: server- IP address: 197.231.94.2

Email viewed by david_wohl@med.unc.edu

2022-06-16 - 2:27:47 PM GMT- IP address: 162.198.202.210

Document e-signed by David Alain Wohl, MD (david_wohl@med.unc.edu)

Signature Date: 2022-06-16 - 2:28:24 PM GMT - Time Source: server- IP address: 162.198.202.210

Email viewed by Robert NSAIBIRNI (robert.nsaibirni@epicentre.msf.org)

2022-06-16 - 2:31:57 PM GMT- IP address: 176.176.79.161

Document e-signed by Robert NSAIBIRNI (robert.nsaibirni@epicentre.msf.org)

Signature Date: 2022-06-16 - 2:32:16 PM GMT - Time Source: server- IP address: 176.176.79.161

Email viewed by Anton CAMACHO (anton.camacho@epicentre.msf.org)

2022-06-16 - 2:58:05 PM GMT- IP address: 176.176.79.161

Email viewed by adebola.olayinka@ncdc.gov.ng

2022-06-16 - 4:48:57 PM GMT- IP address: 197.210.52.11

Email viewed by cmagassouba01@gmail.com

2022-06-17 - 7:32:35 AM GMT- IP address: 197.149.243.96

Email viewed by suzanne.penfold@p-95.com

2022-06-20 - 7:52:56 AM GMT- IP address: 104.47.6.254

Document e-signed by S. C. Penfold (suzanne.penfold@p-95.com)

Signature Date: 2022-06-20 - 9:12:51 AM GMT - Time Source: server- IP address: 85.135.222.150

Signer Anton CAMACHO changed full name at signing to Anton Camacho

2022-06-21 - 10:08:03 AM GMT- IP address: 90.79.246.171

Document e-signed by Anton Camacho (anton.camacho@epicentre.msf.org)

Signature Date: 2022-06-21 - 10:08:04 AM GMT - Time Source: server- IP address: 90.79.246.171

Email viewed by adebola.olayinka@ncdc.gov.ng

2022-06-23 - 3:14:01 PM GMT- IP address: 105.112.227.220

Email viewed by cmagassouba01@gmail.com

2022-06-23 - 3:42:44 PM GMT- IP address: 66.249.93.176

Document e-signed by Bola Olayinka (adebola.olayinka@ncdc.gov.ng)

Signature Date: 2022-06-23 - 3:44:03 PM GMT - Time Source: server- IP address: 105.112.227.220

Email viewed by donkumfel@gmail.com

2022-06-24 - 5:30:26 AM GMT- IP address: 197.215.23.124

Email viewed by cmagassouba01@gmail.com

2022-06-30 - 9:30:36 PM GMT- IP address: 102.129.68.200

Email viewed by cmagassouba01@gmail.com

2022-07-04 - 3:23:36 PM GMT- IP address: 66.249.93.168

Document e-signed by Magassouba N'faly (cmagassouba01@gmail.com)

Signature Date: 2022-07-05 - 4:00:10 PM GMT - Time Source: server- IP address: 197.149.244.251

Email viewed by donkumfel@gmail.com

2022-07-06 - 6:17:22 AM GMT- IP address: 66.249.93.168

Document e-signed by Donald S. Grant (donkumfel@gmail.com)

Signature Date: 2022-07-06 - 6:22:45 AM GMT - Time Source: server- IP address: 197.215.22.96

Agreement completed.

2022-07-06 - 6:22:45 AM GMT
