# Supplementary material for: Correction: A prospective, multi-site, cohort study to estimate incidence of infection and disease due to Lassa fever virus in West African countries (the Enable Lassa research programme)–Study protocol
Source: PLoS One. 2025 Jan 14;20(1):e0317720. doi: 10.1371/journal.pone.0317720 (PMC11731728; doi:10.1371/journal.pone.0317720)
Supplement: S2 File — (PDF) [file pone.0317720.s002.pdf]

Statistical Analysis Plan

PROSPECTIVE MULTI-SITE COHORT STUDY TO ESTIMATE INCIDENCE OF INFECTION AND DISEASE DUE TO LASSA FEVER VIRUS IN WEST AFRICAN COUNTRIES

**SAP version**	Version 4.0, 29th July 2022
**Study Principal Investigators:**	Prof Adebola Olayinka (NiLE Enable)<br>Prof Magassouba N'faly (GUILASSEPI)<br>Dr David Wohl (Liberia Enable)<br>Prof Ayola Akim Adegnika (LAVIHFiB)<br>Dr Donald Grant (Colect Enable)
**Funded by**	CEPI, Coalition for Epidemic Preparedness Innovations, Marcus Thranes gate 2, 0473 Oslo, Norway
**Prepared by**	Epicentre, 14 - 34 Avenue Jean Jaurès, 75019 Paris
**Statistics contact**	Anton Camacho (Epicentre, Paris)<br>Anton.Camacho@epicentre.msf.org

**Revision history**		
Version No	Effective Date	Description
1.0	12-05-2021	First draft of the SAP.
2.0	27-04-2022	Include feedbacks from PHQ partners
3.0	01-07-2022	Change definition of retention and lost to follow-up based on PHQ discussions, correction on appendices
4.0	29-07-2022	Integrate PI's edits

Declaration of Confidentiality

The information contained herein is confidential and therefore are provided in confidence as a potential examiner or investigator.

It is understood that this information will not be disclosed to others without the written permission of the Sponsor, except to the extent necessary to achieve the consent of those who can participate in the study.

Signature Page

Dr Anton Camacho		Aug 27, 2022
Lead Statistician	Epicentre	Signature and Date
Dr Robert Nsaibirni		Aug 18, 2022
Lead Data Manager	Epicentre	Signature and Date
Prof Adebola Olayinka	Adebola Olayinka (Aug 23, 2022 15:40 GMT+1)	Aug 23, 2022
PI Nigeria	NiLE Enable	Signature and date
Dr David Wohl	David Alain Wohl (Aug 18, 2022 09:24 EDT)	Aug 18, 2022
PI Liberia	Liberia Enable	Signature and Date
Prof Ayola Akim Adegnika	aa.adegnika<br>aa.adegnika (Aug 18, 2022 11:07 GMT+1)	Aug 18, 2022
PI Benin	LAVIHFiB	Signature and Date
Dr Donald Grant	Donald S. Grant<br>Donald S. Grant (Sep 19, 2022 18:50 GMT)	Sep 19, 2022
PI Sierra Leone	Colect Enable	Signature and Date
Prof Magassouba N'faly	Prof Magassouba N'faly (Aug 26, 2022 16:41 GMT)	Aug 26, 2022
PI Guinea	GUILASSEPI	Signature and date
Dr Suzanne Penfold	S. C. Penfold<br>S. C. Penfold (Aug 23, 2022 08:18 GMT+2)	Aug 23, 2022
Study Coordinator	P95	Signature and Date
Margaret Williams	Margaret Williams<br>Margaret Williams (Aug 21, 2022 09:28 GMT)	Aug 21, 2022
Founder and CEO of MMARCRO	MMARCRO	Signature and Date

Prof. Stephan Günther	Stephan Günther<br>Stephan Günther (Aug 30, 2022 13:51 GMT+2)	Aug 30, 2022
Head Dept. Virology	BNITM	Signature and Date
Dr Gabrielle Breugelmans		Aug 30, 2022
Director of Epidemiology	CEPI Norway	Signature and Date

Table of Contents

1. Introduction 7
1.1. Statistical Analysis Plan 7
1.2. Rationale 7
2. Study Objectives 8
2.1. Primary Objectives 8
2.2. Secondary Objectives 8
3. Study Design and Assessment 9
3.1. Study Design 9
3.1.1 Study Description and Study Period 9
3.1.2 Inclusion and exclusion criteria 10
3.1.3 Withdrawal/discontinuation criteria 11
3.1.4 Sampling methodology 12
3.1.5 Sample Size and Power Considerations 13
3.1.6 Analysis Populations 15
3.1.7 Protocol deviations and changes to statistical information in the protocol 16
3.2. Study outcome variables 17
3.2.1. Primary outcomes 17
3.2.2. Secondary outcomes 20
3.2.2.1. Secondary outcomes for the LF disease cohort 20
3.2.2.2. Secondary outcomes for the LF infection cohort 23
3.2.3. Other Assessments/Outcomes 24
4. Statistical Analyses 26
4.1 Timing of Analysis 26
4.2 Statistical methodology 28
4.2.1. Primary and secondary outcomes 28
4.2.2. Exploratory analyses 30
4.2.3. Pooling of sites/countries data 30
4.2.4. Methods for Handling Missing Data 31
4.2.5. Confounding factors 33
5. Preparation of tables, listings and figures 34
6 Bibliography 35
7 Appendices 36

Abbreviations

AR	Attack ratio
BNITM	Bernhard-Nocht-Institut für Tropenmedizin
CFR	Case Fatality Ratio
CI	Confidence Interval
CEPI	Coalition for Epidemic Preparedness Innovations
CRF	Case Report Form
CNS	Central Nervous System
EPI	Expanded Program on Immunization
GEE	Generalized Estimating Equations
GLMM	Generalized Linear Mixed Models
GCP	Good Clinical Practice
GPC	Glycoprotein Precursor
GPS	Global positioning system
HH	Household
HHH	Household Head
IgG	Immunoglobulin G
IgM	Immunoglobulin M
IRR	Incidence Rate Ratio
IQR	Interquartile Range
LASV	Lassa Virus
LF	Lassa Fever disease
MI	Multiple Imputation
MICE	Multiple Imputation by Chained Equations
OR	Odds ratio
R&D	Research and Development
REDCap	Research Electronic Data Capture
RDT	Rapid Diagnostic Test
RT-PCR	Real time Polymerase Chain Reaction
PI	Principal Investigator
PP	Per-protocol
PSU	Primary Sampling Unit
SAP	Statistical Analysis Plan
SNHL	Sensorineural Hearing Loss
SOP	Standard operating procedure
SSI	Severity Scoring Index
SSU	Secondary Sampling Unit
STROBE	Strengthening the Reporting of Observational studies in Epidemiology
WHO	World Health Organization

1. Introduction

1.1. Statistical Analysis Plan

The purpose of this statistical Analysis Plan (SAP) is to outline and describe the statistical analyses for the Enable Lassa Research Programme to estimate incidence of infection and disease due to Lassa Fever virus in Western Africa. The project is planned to be carried out in five Lassa Fever endemic countries (Benin, Guinea, Liberia, Nigeria and Sierra Leone) in order to allow an appropriate representation of incidence of LF disease or Lassa Virus (LASV) infection in affected countries in West Africa.

This SAP includes a statement of the objectives of the study, as stated in the protocol; identifies all primary and secondary endpoints; specifies the hypotheses to be tested and any parameters that are to be estimated, to meet the study objectives; defines the analysis populations to be used; and provides a full and detailed description of the methods of analysis including details of handling of missing data, dropouts, etc. All planned analyses and justifications on the methodology are described in the following paragraphs. The document, finally, includes the proposed tables, listings and figures that could be used to present results.

This is a separate document from statistical section of the protocol and was also developed before data was collected. Any changes in the statistical methods presented in the protocol and any additional statistical analysis not included in the protocol will be explained in this detailed statistical analysis plan of the study.
Further details and information on the study can be found in the study protocol version 2.1 from November 2nd 2020 (*cf* protocol)**.**

1.2. Rationale

The Coalition for Epidemic Preparedness and Innovation (CEPI) is funding the development of multiple Lassa vaccine candidates. A crucial step to assess the feasibility and prepare for potential future Lassa vaccine efficacy trials is to gather epidemiological data on the background rates of infection and disease due to LASV in endemic areas, which are needed to assess the sample size requirements of Lassa vaccine efficacy trials. This is necessary because the incidence and spatial distribution of LF is likely to be significantly underestimated based on existing data, due to gaps in diagnostics, surveillance, and access to health services.

The planned prospective multisite cohort study will provide estimates of incidence of infection and disease due to LASV in multiple sites in the countries of interest to inform the design of future vaccine trials and Lassa vaccination strategy when suitable vaccines become available. Conduct of the study will also help to strengthen site and investigator capacity to conduct vaccine trials, as well as to address several gaps identified in the World Health Organization (WHO) Lassa Fever Research and Development (R&D) Roadmap.

2. Study Objectives

2.1. Primary Objectives

- To assess the incidence rate of symptomatic confirmed Lassa Fever disease (LF) cases separately for each of the four Lassa-endemic countries participating in this study with a disease cohort: Benin, Liberia, Nigeria, and Sierra Leone.
- To estimate the incidence rate of LASV infection separately for each of the five Lassa-endemic countries participating in the study: Guinea, Benin, Liberia, Nigeria, and Sierra Leone.

2.2. Secondary Objectives

The secondary objectives are presented in the table below according to the primary objectives they are related to.

General secondary objective	Related to primary objective 1 (LF disease cohort)	Related to primary objective 2 (LF infection cohort)
To Assess Incidence *overall, and by site*	Incidence rate of symptomatic confirmed LF	Incidence rate of LASV infection
To assess Incidence *overall, by country, and by site*	Age-specific incidence rate of symptomatic confirmed LF	Age-specific incidence rate of LASV infection
		Age-specific incidence proportion of seroreversion <u>over time</u> (except baseline)
	Incidence rate of 'acute febrile illness'	
	Monthly Incidence rate of symptomatic confirmed LF	
To assess prevalence *overall, by country, and by site*	Baseline seropositivity prevalence	Baseline seropositivity prevalence
		Age-specific seropositivity prevalence at baseline and <u>over time</u>
		Age-specific seroreversion period prevalence <u>over time</u> (except baseline)
	Prevalence of lab confirmed LF among 'acute febrile illness' cases.	
	Prevalence of symptomatic confirmed LF co-infected with malaria parasites	
To assess the role of selected risk factors	Role of selected risk factors for symptomatic confirmed LF disease *overall and by country*	Role of selected risk factors for LASV infection at baseline and <u>over time</u>*: overall, by country, and by site*
Other objectives specific to the LF disease cohort and that will be presented	To assess the association between baseline seropositivity and the occurrence of symptomatic confirmed LF	

overall, by country, and by site	To describe the clinical course of symptomatic confirmed LF	
	To determine the case fatality rate (CFR) among symptomatic confirmed LF cases	
	To determine the proportion of Sensorineural Hearing Loss (SNHL) among patients with symptomatic confirmed LF	
	Determine the proportion of LF survivors patients with delayed or persistent SNHL	
Other objectives specific to the LF disease cohort	To describe clinical course and outcome of symptomatic confirmed LF cases stratified by risk group: *Pregnant women, children and elderly*	
	To define different levels of severity of symptomatic confirmed LF cases	

Table 1: Secondary objectives according to the primary objective they are related.

3. Study Design and Assessment

3.1. Study Design

3.1.1 Study Description and Study Period

This is a prospective multi-site cohort study designed to assess the incidence rate of symptomatic LF cases through the LF disease cohort; and to estimate the incidence rate of LASV infection through the LASV infection cohort. Symptomatic LF cases are to be confirmed by Polymerase Chain Reaction (PCR) and LASV infection to be assessed by seroconversion.

In the four sites where both LF disease and LASV infection cohorts are implemented (Edo site in Nigeria, Benin, Sierra Leone and Liberia sites), the LASV infection cohort (n = 1000) will be nested in the LF disease cohort (n = 5000). A total of 7 sites across the five countries of interest will be involved in the study, with Nigeria having three sites, and the four remaining countries having each one site.

Recruitment start will depend on sites readiness as per protocol (PP). It is noteworthy that recruitment was supposed to be done outside of the high LASV transmission season (preferred recruitment period is from end of March to end of November). In case of delayed start of the study recruitment, potential overlap with the high LASV transmission period should be accounted for in the analysis and further discussed since it could lead to overestimation of the baseline seroprevalence and under-estimation of infection and symptomatic incidence. Such a scenario might also require an extension of the study period at the sites with delay so that participants could be followed for two full Lassa seasons.

At baseline, participants are asked for informed consent and confirmation of eligibility. All study participants are planned to be followed over a period of 12 (minimum) to 24 (maximum) months. Participants in the LF disease cohort will be followed by an active surveillance system with 2-week (14-day) follow-up and passive surveillance, whereas participants in the LASV infection cohort will be

followed every 6 months from baseline (until the maximum 24-month follow-up) for blood sample collection. (Figure 1). Disease cohort follow-up can be done by telephone or in person, on a rotating basis but with varying ratios phone/in-person depending on the human resources available at each site. At any time of the study, the participant can withdraw their consent and terminates the study.

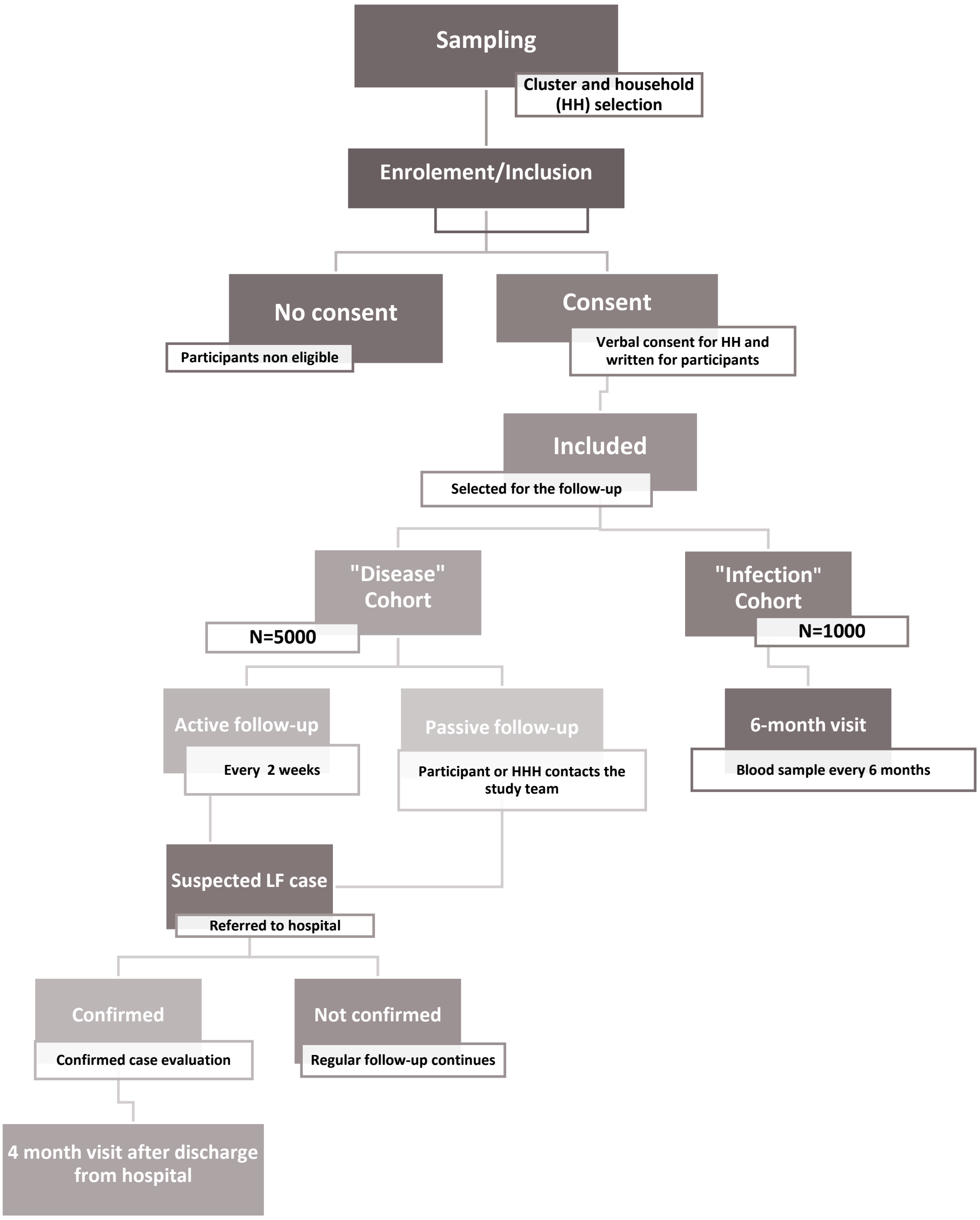

Figure 1: General procedure diagram

3.1.2 Inclusion and exclusion criteria

Subjects failing to meet inclusion criteria and those who meet exclusion criteria at screening visit will not be included to either of the two study cohorts. Study inclusion and exclusion criteria are listed in Table 2.

In all countries, the head of the household decides whether the entire household will participate by giving a verbal consent, without excluding any particular members.

Inclusion criteria	Exclusion criteria
• Healthy females and males over 2 years of age	• Persons unwilling to comply with any of the study procedures, including blood specimen collection;
• Resident of the study area for six months preceding recruitment, and expected to stay in the area for most of the time until the end of the first LASV high transmission season (but also depending on recruitment start)	• Persons indicated by the respective household head as not eligible
• Able and willing to provide informed consent (and assent, as required), according to country specific procedures	• Any individual who for medical or social reasons cannot be enrolled in the study
• Able to understand the language (official language of the country or local languages) or have a surrogate available who can translate	• Persons who may not be able to consent freely, such as persons in military service
• Household head (HHH) has granted permission for household (HH) member to be approached by research team (if necessary)	• Any other reason which in the discretion of the study personnel would interfere with a person's ability to participate in the study (e.g., mental illness)
• Suitable measures for future study contacts via household head and secondary household contact person are in place (mobile phone, fixed telephone number)	• Any study staff and study site personnel (relatives of study staff are not excluded)

Table 2: Inclusion and exclusion criteria

As indicated in this table, the second inclusion criterion precises participant should stay in the study site area for most of the time until the end of the 2020-2021 LASV high transmission season. Given some countries/sites will start inclusion after that time window, this criterion will then be adapted to the time of study start.

3.1.3 Withdrawal/discontinuation criteria

The participants may be withdrawn from the study for any of the following situations:

- Participant or their parent / legal guardian (if appropriate) wishes to withdraw consent,
- Participant or household migrates from the study area permanently,
- Participant is lost to follow-up,
- Participant falls ill **or** dies,

- Participant is no longer interested in study,
- Participant no longer wants to give blood,
- Participant states being too busy to continue study.

In all such cases, the participant will be withdrawn from the study and the reason for withdrawal will be documented (if offered) in an appropriate case report form (CRF)-Study termination CRF N.11- and listed (Listing 1.2) for further inquiries. All subjects, who withdraw early from the study for any reason, will be encouraged to complete the study termination assessment and if completed personally and authorization given, all information already collected up to the last contact will be retained for analysis.
As per protocol, withdrawal of consent is possible for participants at any time in the study and will have no effect on their medical care or access to treatment.
Subjects who move away from the study site or are lost to follow-up (miss two consecutive active follow-up visits where at least three attempts were made for each) and later present to the site while study is still ongoing, and are willing to continue participation, will continue to be followed through the end of planned follow-up (i.e. no additional visits are added to compensate the missed visits). In such case, the participant contact log form (CRF N.2) will be filled again to give information on the reintegration of the study. In Liberia, it was decided not to have a lost-to follow-up case definition. This means that attempts to contact participants will continue for the duration of the planned active follow-up, regardless of when the last contact was made.

3.1.4 Sampling methodology
Ideally, all five countries would have used the same sampling methodology as PP, however this was not possible for practical reasons and inherent specificities of each setting.

First, the choice of the districts/regions to include in the study was each country's responsibility and detailed in their specific adapted protocols (if available). The definition of the study population varied across countries, with some countries focusing on Lassa hotspot communities while other on larger administrative areas.

Second, although the study protocol suggested to use the sampling method of the WHO's Expanded Program on Immunization (EPI) to select households within each cluster, this methodology does not generate a probabilistic sample and alternative approaches were discussed with each country [1]. Eventually, the methodology chosen was a two-stage sampling with differences between countries in the method of selecting the primary sampling unit (PSU) which were the localities (villages) and the secondary sampling unit (SSU), which were the households (HH). Similarities between members of the same HH and among inhabitants of a village induce clustering effects and it was recommended to the sites to use as many clusters (localities/villages) as feasible to reduce the design effect of the study.

---

[1] TURNER, MAGNANI, and SHUAIB, "A Not Quite as Quick but Much Cleaner Alternative to the Expanded Programme on Immunization (EPI) Cluster Survey Design."

The national authorities in each site provided demographic data on the population of the selected villages/communities (cluster size) as well as household enumeration for each cluster. In some cases, it was difficult to obtain up-to-date data from national authorities due to outdated censuses or changes between the latest available census data and the current administrative division. In such cases, we used data from recent public health programs (e.g. bed-net distribution campaigns or mass drug distribution) that conducted micro-censuses in the targeted communities. Weights for each cluster were then derived from the population data, and the number of people to be recruited was calculated for each cluster.

Study population: Nigeria, Liberia and Guinea selected a limited number of Lassa hotspot communities based on previous history of Lassa cases, presence of rodents and/or proximity with the Lassa reference center. By contrast, Benin and Sierra Leone selected larger rural administrative areas containing hundreds of localities/villages, some of which reported cases of Lassa in previous years.

Cluster selection: For Nigeria, Liberia and Guinea, the clusters were purposively sampled and correspond to the communities selected for the study population. By contrast, in Benin and Sierra Leone, a limited number of localities/villages were randomly sampled in the study population. In Benin, clusters were sampled proportionally to the size of the population. In Sierra Leone, administrative sections were sampled proportionally to their size and then we randomly sampled a given number of villages within each section.

Household selection: Nigeria and Guinea used a systematic sampling to select households from exhaustive enumeration lists obtained for each cluster. Liberia and Benin used a satellite-imagery approach to randomly sample GPS coordinates corresponding to households [2]. Finally, Sierra-Leone decided to include all households of the selected villages, for the sake of population acceptability and based on their previous experience in the study area.

Further details on the sampling methodology for each country are provided in an external document referred as SAP-**Appendix 1**.

3.1.5 Sample Size and Power Considerations

This paragraph is extracted from the study generic protocol and explains the methodology used by P-95 colleagues for the sample size calculation. The sample size was estimated for each cohort (infection and disease) in a given site. In countries with multiple sites, the final sample size is the sum of the sample size in each site.

To guide the sample size calculation of the disease cohort, estimations were made for different precision levels considering the incidence of LF to be between 0.001% and 3%. For an incidence rate of 0.1% over the two-year period (or 1/1,000), the precision would range from 0.04% (or 0.4/1,000)

[2] Miller et al., "Feasibility of Satellite Image and GIS Sampling for Population Representative Surveys."

to 0.23% (or 2.3/1,000) as presented in table 1. For an incidence rate of 1% over the two-year period, the precision would range from 0.76 % to 1.32%.
It was then estimated that 5,000 subjects were needed to be recruited per country (or per site per country in those with multiple sites) to estimate the incidence of symptomatic infections with a 95% CI of 7.6 – 13.2 for an incidence of 10/1,000 person-years or a 95% CI of 0.4 – 2.3 for an incidence of 1/1,000 person-years.

Table 3-1: Sample size estimates – Incidence rate (%: per 100 persons over 2-year period)

**Sample size**	**N = 200**		**N = 1 000**		**N = 5,000**		**N = 25 000**	
**Incidence rate (in %)**	Lower 95 % CI	Upper 95 % CI	Lower 95 % CI	Upper 95 % CI	Lower 95 % CI	Upper 95 % CI	Lower 95 % CI	Upper 95 % CI
**0,001 %**	0,00	1,89	0,00	0,39	0,00	0,08	0,00	0,02
**0,01 %**	0,00	1,90	0,00	0,40	0,00	0,10	0,00	0,03
**0,1 %**	0,01	2,08	0,02	0,56	0,04	0,23	0,07	0,15
**1 %**	0,28	3,57	0,54	1,83	0,76	1,32	0,84	1,13
**3 %**	1,38	6,39	2,11	4,25	2,56	3,51	2,80	3,22

Confidence intervals (lower and upper limits 95%CI) were calculated using the Wilson method.

The same methodology was used for the sample size calculation of the infection cohort with calculations made for different precision levels (considering seroprevalence rate between 1% and 50%, table 2). To estimate the incidence of LASV infection with a 95% CI of 0.5 – 1.8 for an incidence of 1/100 person-years or a 95% CI of 8.3 – 12.0 for an incidence of 10/100 person-years, 1,000 subjects were deemed necessary to recruit per country (or per site per country in those with multiple sites).

Table 3-2: Sample size estimates – Seroprevalence rate (%: per 100 persons)

**Sample size**	**N = 200**		**N = 1 000**		**N = 5,000**		**N = 25 000**	
**Seroprevalence rate (in %)**	Lower 95 % CI	Upper 95 % CI	Lower 95 % CI	Upper 95 % CI	Lower 95 % CI	Upper 95 % CI	Lower 95 % CI	Upper 95 % CI
1 %	0,3	3,6	0,5	1,8	0,8	1,3	0,9	1,1
5 %	2,7	9,0	3,8	6,5	4,4	5,6	4,7	5,3
10 %	6,6	14,9	8,3	12,0	9,2	10,9	9,6	10,4
50 %	43,1	56,9	46,9	53,1	48,6	51,4	49,4	50,6

Confidence intervals (lower and upper limits 95%CI) were calculated using the Wilson method

The sample size did not consider the baseline seropositivity rate. If the seropositivity rate in the general population is high, this would likely diminish the pool of susceptible individuals and decrease the incidence rates of LF. Sites were encouraged to consider ways of mitigating this issue by

considering ways of enrolling more subjects if high seropositivity rates were to be observed at baseline. Sites could also consider recruiting participants from a different geographic area. An assessment will be made during the interim analysis.

An important limitation of the sample size calculation was the omission of the clustering effect within the study design (at cluster and household level), assuming statistical independence among participants. As such, the confidence intervals on the incidence and prevalence specified in the protocol (in table 3-1 and 3-2) are likely narrower than those that will eventually be obtained from the analyses after accounting for the clustering of the participants.

3.1.6 Analysis Populations

The number of participants in each of the following analysis populations, along with number and percentage of participants included to each cohort, will be provided.

The **LF disease cohort** will include all participants that were included as per eligibility criteria and randomization methodology to this cohort.
In sites implementing both cohorts, the **LF infection cohort** will include all participants from the LF disease cohort chosen to be also part of the nested cohort (LF infection). They are randomly selected from the disease cohort in a one in every five households during the enrolment. Thus, in Benin, Liberia, Irrua (Nigeria) and Sierra Leone, the LF disease cohort population includes the LF infection cohort population.
The "**acute febrile illness**" or **suspected case** classification concerns any participant who self-reported fever of more than 48 hours duration (lasting at least two consecutive nights) **and**:

- who has a history of contact with a confirmed LF case,
  OR
- who presents one of the signs/symptoms listed in the following table:

• Headache	• Sore throat	• Jaundice
• Chest pain	• Abdominal pain	• Spontaneous abortion
• Muscle or joint pain	• Abnormal bleeding (from mouth, nose, rectum and/or vagina)	• Buzzing in ears or acute deafness
• Vomiting	• Oedema of the neck and/or face	• Hypotension
• Cough	• Conjunctival or sub-conjunctival haemorrhage	

Table 3: list of signs and symptoms associated with Lassa fever disease.

The **confirmed LF cases** population will include all participants presenting an acute febrile illness (defined above) and who have a positive Lassa RT-PCR result.
The **seroconverted** population is made of all participants with a documented change from no detection of immunoglobulin G (IgG) antibodies (seronegative) to either their detection (seropositive IgG+ status) or detection of IgM (IgM+ status); through serology tests at two distinct time points.
The **seroreverted** population is made of all participants with a documented change from seropositive to seronegative status (through serology tests at two distinct time points).
The **confirmed LF cases with fatal outcomes** population include all *confirmed LF cases* who die within 30 days of diagnosis or whose death was attributed to LF disease as assessed by the treating clinician at any point following LF confirmation.
SNHL-affected participants are those diagnosed with a hearing loss of at least 30dB in three sequential frequencies in the standard pure tone audiogram, where a physical examination has excluded conductive hearing loss.
The **LF survivors with delayed SNHL** population include all *confirmed LF cases* discharged from hospital with an audiometry consistent with SNHL at follow-up but not at hospitalization.
The **LF survivors with persistent SNHL** population include all *confirmed LF cases* discharged from hospital with an audiometry consistent with SNHL at follow-up and at hospitalization.
Paediatric participants are all participants under the legal age according to the country.
Elderly population are all participants above *65 years old*.
Pregnant women population include all confirmed LF women cases admitted to hospital and confirmed or reported pregnant. It is important to highlight here that information on pregnancy test is only collected either at hospital admission or at the time of discharge from hospital.
Retained participants in the disease cohort are participants who completed the last planned active follow-up visit (second visit of the 24th month of follow-up). It is noteworthy that follow-up is planned to continue for confirmed cases even after the 4-month post discharge visit.
Retained participants in the infection cohort are participants who complete the fourth 6-month follow-up visit (regardless of whether they had seroconverted).
Lost-to-follow-up (LTFU) participants in the disease cohort are participants in the disease cohort who missed at least two consecutive visits and remained unseen until the planned end of the study.
Lost-to-follow-up participants in the infection cohort are participants who missed at least one follow-up visit and remained unseen until the planned end of the study.
Temporary LTFU participants are participants in the disease cohort who missed at least two consecutive visits but had at least another subsequent successful visit before the end of the study. In the final analysis, they will be referred to as participants with incomplete follow-up.

3.1.7 Protocol deviations and changes to statistical information in the protocol

Any changes from protocol-specified procedures and study-related SOPs occurring during the conduct of the study will be documented and reported as protocol violations (major deviations) or minor deviations, as defined in the protocol. A final Protocol Deviation Listing will be generated (Listing 5.1 and 5.2) and reviewed blindly by the sponsor prior to freezing the database to ensure that all

important deviations, including those that may lead to exclusion from analysis, are captured and summarized.

- Participants will be excluded entirely from analysis if they have a protocol deviation defined as a full exclusion that affects the validity of their data (e.g., failure to obtain informed consent).
- Participants with a minor protocol deviation (e.g., moving permanently away from study area) will be included up until the time of the deviation; from this point onwards, their data will be excluded from analysis (except the case when they re-enter study).
- If a deviation only affects data at a specific time point (e.g., blood sample not taken within defined time window), only data affected by the deviation will be removed from analysis.

For analyses, if any of the statistical methods proposed at the time of writing this SAP are found to be unsuitable for the final analysis, more appropriate alternatives will be used. All such changes and the rationale for their use will be documented by amending this SAP.

3.2. Study outcome variables

3.2.1. Primary outcomes

The primary outcome for both cohorts is based on the incidence rate defined as the number of new LF cases or new LASV infections divided by the total person-time-at-risk for the cohort population. The denominator of this formula (the person-time) will be expressed in years and is the total amount of time that the study participants are at risk of becoming a confirmed LF case or seroconverting. It will be calculated by the total amount of time participants remain in the study (from baseline) until one of following “end points” is reached:

- onset of LF disease or seroconversion, depending on the cohort,
- first classification as temporary lost-to-follow-up if incomplete follow-up and no onset of LF disease by the end of the study,
- completion of the study (planned end of study),
- early study termination (due to death, **permanent** migration outside the study area, consent withdrawal…).

Participants are therefore eligible to contribute to the study person-years only if they are still at risk of becoming a confirmed case (disease cohort) or seroconverting (infection cohort). In other words, even in the case of missed follow-up visits, as long as we can “assume” that the participant has not yet experienced the outcomes of interest, we increment the time-at-risk (s)he contributed to the study. Note that we do expect participants to miss several visits (temporary lost to follow-up) and re-integrate study (in countries where LTFU is considered).
Any participants that are permanently lost to follow-up or terminated early prior to experiencing LF disease or seroconversion (depending on the cohort) will be censored at the day of their last observed assessment (last visit or date of consent withdrawal). Subjects who complete follow-up but do not experience the disease or seroconversion (depending on the cohort) will be censored at the day of their last follow-up visit.

- LF disease cohort

Incidence rate of confirmed symptomatic LF: number of *confirmed LF cases* per 1,000 person-years of follow-up in the symptomatic disease cohort over the entire study period, for each country.

$$\text{Incidence Rate}_{\,i} = \frac{\#\, new\ participants\ positive\ to\ RT - PCR}{total\ person - time - at - risk\ (in\ years)}$$

With "*i*" the countries of interest: Benin, Liberia, Nigeria (Irrua site), and Sierra Leone.
With regard to the construction of the denominator for the calculation of the incidence rate in the disease cohort, ignoring the absence of a participant who re-enters the study at a later time (after a temporary LTFU) may overestimate the person-years and thus underestimate the incidence rate. In fact, if a participant migrates from study area for a sufficient period of time to develop LF and be cured, re-entering that participant after his or her return would make his or her contribution to the study longer than it actually is, since we have no way of verifying that he or she has contracted LF and would continue to consider him or her to be at risk. For this reason, although participants may re-enter the study after a temporary break, if they did not experience the disease until the end of follow-up, we will consider their exit time as the last time point before temporary migration or loss to follow-up in this analysis (censoring). However, if the participant were temporary lost then reintegrate study and develop the disease prior to end of follow-up, their entire follow-up time will be kept in the analysis.

- LASV infection cohort

Incidence rate of LASV infection: number of LASV infections (seroconversion) per 1,000 person-years of follow-up in the infection cohort over the entire study period, for each country.

$$\text{Incidence Rate}_{\,i} = \frac{\#\, new\ participants\ who\ seroconverted}{total\ person - time - at - risk\ (in\ years)}$$

With "*i*" the countries of interest: Guinea, Benin, Liberia, Nigeria (all 3 sites), and Sierra Leone.

In calculating person-years for the infection cohort, it is important to estimate the date of seroconversion correctly. Therefore, midpoint imputation will be used, which means that

seroconversion is assumed to have occurred halfway between the baseline visit and the first 6-month follow-up visit or between two 6-month follow-up visits. As per protocol, only IgG status will be assessed and documented at baseline and any study participants found to be IgG+ will be considered seropositive at baseline. At the four 6-monthly follow-up occasions both IgG and immunoglobulin M antibodies (IgM) will be documented and interpreted as follows:

**Serology** (anti-LASV)	**Cut-off**
IgG-	< 1.0 µg/mL
Indeterminate	[1.0 - 1.8[µg/mL
IgG+	≥ 1.8 µg/mL

Table 4: Serology results cut-off for IgG ELISA

**Previous Serology**	**Current Serology**	**Interpretation**	**Details on interpretation**
IgG- →	IgG+	LASV infection (timing according to IgM)	**Seroconversion** in a presumably Lassa virus naive (never exposed) person.
Ab concentration ≥ 1.0 µg/mL and not in the saturation part of the calibration curve	Ab concentration with at least 4-fold increase from previous serology and not in the saturation part of the calibration curve	Booster infection in previously exposed person	This interpretation must be considered speculative and should be presented as "-fold increase in dilutions steps" and no other dimensions (i.e. concentrations).
IgG+ →	IgG-	LASV reversion	**Seroreversion** in a person previously exposed to Lassa virus.
IgG+ →	IgG+, With < 4-fold increase	*None, IgM results needed*	- If IgM+ then could be recent infection
IgG- →	IgG-	*None, IgM results needed*	- If IgM+ then could be recent infection
Indeterminate	IgG+ With < 4-fold increase	*None, IgM results needed*	- If IgM+ then could be recent infection

Indeterminate	IgG-	*None, IgM results needed*	- If IgM+ then could be recent infection

Table 5: Interpretation of serology at 6-month follow-ups

The laboratory responsible for evaluating the performance of the immunoassays suggested that seroconversion should be based on the existence of a change in concentration from below the lower cut-off to above the upper cut-off value, as the assay provides the Ab concentration.
For indeterminate results (Ab concentration between 1.0 and 1.8 µg/mL), it is suggested that they be considered presumptively positive. However, if the indeterminate result is closer to the lower cut-off, the sample should be retested or a new sample should be taken for testing. In the event that retesting is not possible, any result below 1.4 µg/mL can be considered presumptively negative.

3.2.2. Secondary outcomes

All the secondary outcomes below will be presented for both the pooled cohort (overall) and separately for each county or site (whichever is applicable).
Please note that all countries where the disease cohort is implemented have a single site involved, so the following outcome measures stratified by study site will be the same as those stratified by country.

3.2.2.1. Secondary outcomes for the LF disease cohort

Incidence rate of confirmed symptomatic LF:

- The overall number of *confirmed LF cases* in the symptomatic disease cohort per 1,000 person-years of follow-up will be calculated via pooling of the data of the different geographic-endemic areas under study.
- All countries where the disease cohort is set up have one site involved, hence the incidence stratified by study site corresponding to the country estimated incidence rate (primary objective).
- The overall incidence rate will then be stratified by gender, baseline serostatus and participant age-groups defined as following: <5, 5-17, 18-24, 25-34, 35-44, 45-54, 55-64, >65 (years old) or organized in larger groups.
- The overall number of *confirmed LF cases* in the symptomatic disease cohort per 1,000 person-years of follow-up will be calculated via pooling of the data of the different geographic-endemic areas under study.

Clinical course of LF:

- The percentage of *confirmed LF cases* presenting clinical signs and symptoms, requiring certain interventions and complications, among all confirmed LF cases. This will refer to the percentage of confirmed cases classified with a disease stage 3 or 4 (upon admission, during admission and just before discharge) with all symptoms from the stages 3 and 4 considered

as complications (see Table 6). CRF will not inform on the clinical stage but based on symptoms information filled into ***REDCap***, the clinical stage classification will be done during the analysis phase using the LF clinical stage from Richmond *et al*[3]**.**

If several symptoms typical of different stages are present at the same time for a time point, the symptom of the highest stage will inform the stage assigned to the patient at that time point.

Stage	Symptoms	Days since onset
**1**	General weakness and malaise. High fever, >39°C	Days 1-3
**2**	Sore throat (with white exudative patches) very common; headache; back, chest, side, or abdominal pain; conjunctivitis; nausea and vomiting; diarrhoea; productive cough; low blood pressure (systolic <100 mm Hg); anaemia	Days 4-7
**3**	Facial oedema; convulsions; mucosal bleeding (mouth, nose, eyes); internal bleeding; confusion or disorientation	>7 days
**4**	Coma and death	> 14 days

Table 6: Clinical stages of Lassa Fever Disease (adapted from Richmond et al. 2003)

The relevant clinical signs and symptoms and their corresponding complications are described in the table below selecting variables from the Suspected case evaluation and/or the Data extraction forms.

- The previous outcome will be presented overall and stratified upon relevance by the following risk groups:
  - Pregnant women
  - Children below 5 years old
  - Immunocompromised patients (HIV…)
  - Patients with NCDs (Diabetes, Kidney diseases, etc.)

LF Clinical stage	Signs/Symptoms	Risk group involved
**1**	General malaise	All
	Fever	All

[3] Richmond and Baglole, "Lassa Fever."

**2**	Conjunctivitis	All
	Headache	All
	Diarrhoea	All
	Chest pain	All
	Muscle or joint pain	All
	Vomiting	All
	Cough	All
	Sore throat	All
	Abdominal pain	All
	Hiccups	All
	Rash	All
	Anaemia	All
	Hypotension	All
**3**	Abnormal bleeding (from mouth, nose, rectum and/or vagina)	All
	Internal bleeding	All
	Oedema (swelling) of the neck/face and/or leg/feet	All
	Conjunctivital or sub-conjunctivital haemorrhage (red eyes)	All
	Jaundice	All
	Spontaneous abortion	Pregnant women
	Buzzling in ears	All
	Acute deafness	All
	Seizures/convulsion	All
	Confusion/disorientation	All
	Dizziness	All
	Renal/Liver failure	All
**4**	Coma	All
	Death	All

Table 7: Lassa fever disease sign and symptoms by clinical stage and risks groups concerned by each

The **Case fatality ratio** will be calculated as follows:

$$CFR = \frac{\#LF\ cases\ dying\ within\ 30\ days\ of\ diagnosis\ or\ attributable\ to\ LF\ disease}{\#LF\ cases\ with\ known\ outcome\ at\ the\ end\ of\ study}$$

and defined as the percentage of *confirmed LF cases* dying within 30 days of diagnosis or attributable to LF disease as assessed by the treating clinician at any point post confirmation. It is important to exclude from the denominator the confirmed cases with unknown outcomes to avoid underestimation of the CFR.

Prevalence:

- **of symptomatic confirmed LF co-infected with malaria parasites:** percentage of all *confirmed LF cases* among whom presence of malaria parasites assessed by antigen rapid diagnostic test (RDT) is detected at the time of LF diagnosis.
- **of symptomatic confirmed LF among all 'acute febrile illness':** percentage of all participants classified as 'acute febrile illness' (see analysis population definitions) who were lab-confirmed for LF disease.
- **Baseline seropositivity prevalence**: percentage of all participants in the disease cohort classified as seropositive at baseline.

Occurrence of SNHL or other sequelae:

- Percentage of all *confirmed LF cases* with SNHL assessed by audiometry prior to discharge.
- Percentage of all LF survivors with delayed SNHL at 4 months after hospital discharge.
- Percentage of all LF survivors with persistent SNHL at 4 months after hospital discharge.
- In addition to audiometry, there will be an open question enquiring about the health status of the study participant to ask whether they are suffering from any other sequelae. Thus, the percentage of all LF survivors with any other sequelae at 4 months after hospital discharge will be also calculated.

Assessment of risk factors for symptomatic confirmed LF: Incidence rate ratios (IRR), which are the ratio of the incidence rates calculated in the prespecified risk groups to the incidence rate in the risk-free group, and their 95% CI will be presented to assess the association between the incidence rate of symptomatic LF disease and characteristics of the study subjects (being a male, being a child or an elderly, being a healthcare worker, consuming rodent meat…), as listed in the questionnaires (form 1) and listed in the table below:

Participant characteristics	Household characteristics
Age	Presence of rodents
Sex	Sight of rodents
Occupation	Consumption of rodent meat
Contact/sight of rodents	History of LF for a HH member
Previous diagnosis of LF	Presence of a HCW in the HH
	Low storing of food
	Type of housing
	Presence of ceiling

Table 8: Participants and household baseline characteristics

3.2.2.2. *Secondary outcomes for the LF infection cohort*

Incidence rate of LASV infection:

- The overall number of LASV infections (seroconversion) per 1,000 person-years of follow-up will be calculated via pooling of the data of the different geographic-endemic areas under study.
- The number of LASV infections per 1,000 person-years of follow-up will be then estimated by site for Nigeria.
- The other countries where the infection cohort is set up have one site involved, hence the incidence stratified by study site corresponding to the country estimated incidence rate (primary objective).
- The overall incidence rate will then be stratified by participant age-groups defined as following: <5, 5-17, 18-49, 50-79, >79 (years old).

Incidence proportion of seroreversion:

- The age-specific number of LF cases who newly seroreverted among those at risk using the age-groups defined previously, will be estimated at the 4 timepoints after baseline and calculated as:

$$Incidence\ i = \frac{\#new\ seroreversions\ at\ 6 - month\ visit\ i}{\#seropositives\ at\ 6 - month\ visit\ i - 1}$$

Prevalence:

- **Baseline seropositivity prevalence:** the overall percentage of subjects found to be seropositive at baseline (IgG+), (as described previously) out of the number of subjects tested for baseline seropositivity. It will then be presented for each of the age-groups previously described.
- The **seropositivity prevalence** will be also calculated and presented for each of these age-groups over the 4 others different timepoints in addition to the baseline visit.
- **Seroreversion prevalence**: the overall percentage of 'seroreverted' participants (as described in 3.1.6 section) out of the number of participants who were seropositive at the previous timepoint for each of the age-groups previously described.

Assessment of risk factors for LASV infection: IRR will be used in the same way as for the risk factors for the symptomatic LF to assess the association between the incidence rate of LASV infection and the same characteristics as aforementioned (Table 8).
The IRR will be also calculated over the timepoints (follow-up visits) other than baseline.

Assessment of factors associated with baseline seropositivity: the contribution of certain conditions (pregnancy, young age, elderly) to baseline seropositivity will be assessed by estimating the ratio of the odds of being seropositive at baseline in prespecified risk groups to the odds in the study population without the prespecified risk. As a result, it will be expressed as an odds ratio (OR).

3.2.3. Other Assessments/Outcomes

Besides primary and secondary outcomes, additional exploratory outcomes have been thought relevant for the study and will be described briefly in this document and planned to be undertaken for the final/primary analysis.

Since a high **retention rate** in longitudinal studies plays a crucial role in preventing potential biases due to missing data, retention rates within sites will be estimated for each country. The retention rate is defined as the number of individuals who remained in the study at the last wave of data collection as a proportion of the total number of participants recruited at the baseline assessment. As indicated in the analysis populations (3.1.6), **retained participants** are those who completed the last bi-weekly active follow-up visit (disease cohort) or the last 6-month visit (infection cohort), 24 months post-enrolment.
We will also evaluate baseline characteristics that predict study retention.

Overall attack rates within sites are planned to be estimated based on data of full season to assess which population is the most at risk. The crude attack ratio (AR) of a disease is a measure of morbidity defined as the number of new cases divided by the population at risk. The population at risk in this case is the number of those susceptible to the circulating virus type(s) before the start of the first LF season:

$$AR = \frac{\text{Number of cases}}{\text{Population at risk}}$$

The attack rate will be calculated in each country for several sub-populations and different follow-up periods according to the particularities of each country.

Household attack-rate: since the sampling was done at the household level and all eligible and consenting household members were enrolled in the study, we will investigate the intra-household disease attack rate in every household with at least one confirmed Lassa case. In households that were included in both disease and infection cohorts, we will also calculate the intra-household infection attack-rate.

Severity scoring index (SSI) for LF has not yet clearly been determined, yet studies performed in Nigeria have previously assessed demographic, clinical, and laboratory factors associated with LF fatal outcome. Findings have highlighted acute kidney injury (AKI), severe central nervous system (CNS) symptoms such as coma, seizure; irrational behaviour, jaundice, bleeding, and non-severe CNS symptoms (dizziness, lethargy, drowsiness) as clinical features with enough evidence of association with LF death[4]. To verify or confirm such findings, modelling is planned in the exploratory analyses to address mainly two specific and relevant questions: analysis of the risk related to severity and of the

[4] Okokhere et al., "Clinical and Laboratory Predictors of Lassa Fever Outcome in a Dedicated Treatment Facility in Nigeria."

risk of fatal outcome related to the evolution of biological measures (blood chemistry and blood counts collected in the data extraction form and extracted from medical records).
Based on our results, a proposal for a SSI for LF can be made if satisfactory results are obtained. This information will be valuable for the clinical management of LF cases in future epidemics.

For the population of confirmed LF cases, an aliquot will be collected after confirmation by RT-PCR for genome sequencing. Genetic analyses, such as the construction of phylogenetic trees, will then be carried out to fill in the important gaps that remain in the phylogeography of Lassa virus, both at national and supra-national levels.

Since the infection cohort is embedded in the disease cohort, we also plan to combine some of the analyses e.g by looking at symptomatic infection among all infection and how some prespecified risk factors contribute to the fact of being symptomatic. In such analyses we would pool data from all countries/sites (except Guinea, and Nigeria's *Owo* and *Abakaliki* sites).

Additional modelling is planned to be undertaken separately for each country to better understand the **dynamics of Lassa Fever outbreaks**. Dynamical models can integrate both incidence and seroprevalence data to better understand the geographic and temporal dynamics of the outbreaks and the mechanisms shaping the transmission of the virus. Our aim is to set an inference framework that can integrate our study data with the national surveillance system, as well as other relevant datasets such as climatic data (rainfall). Regarding seroreversion, to go deeper into the analysis, a model for time to seroreversion will be explored using serostatus as binary outcome or if data makes it possible using continuous titre values.

For some countries/sites, additional data is being collected mainly on pregnant women for Benin, PCR tests for the infection cohort in Guinea, and additional biological data on urine and blood in Liberia. Analyses related to these non-core research objectives will not be presented in this SAP and may require contacting the relevant implementing partner directly.

4. Statistical Analyses

4.1 Timing of Analysis

A complete interim analysis is planned to be undertaken at the end of the first full Lassa season according to the start of the study in each country. The main purpose of this interim analysis is to analyze data collected after the first full Lassa season for the primary objectives and selected secondary objectives (defined in the section below). The aim is to assess the probability that the continuation of the study during the second season will provide additional knowledge on the first primary objective.
Given the different study start dates, the timing of the interim analysis, as well as other planned analyses will differ between countries. In fact, the delay in the enrollment starts of participants (from the period planned in the protocol) affects the completeness of a full Lassa Fever season and makes

an interim analysis possible only by the spring of 2021 for Nigeria. For the other four countries a longer delay may be expected.

The final analysis could be then expected during spring 2023, at the earliest, to allow for a minimum of 12 months follow-up to all participants that would not prematurely terminate study. The figure below gives an idea of the possibility of such an extension and how it will affect timing of analysis.

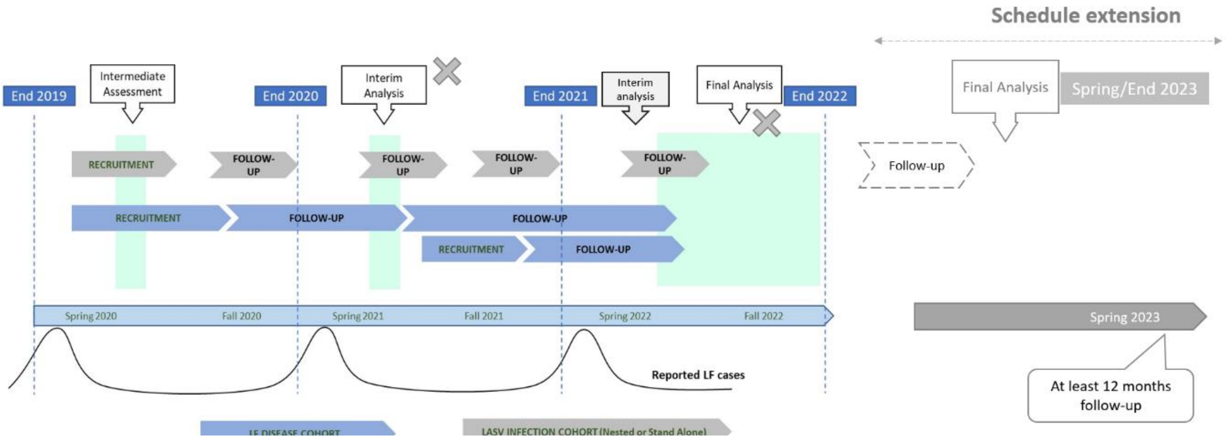

Figure 2: Interim analyses schedules

Another issue regarding the analyses timing concerns the beginning of the enrollment during the epidemic season in some countries, with a risk of under-estimation of the LF incidence in the different countries. In Nigeria for instance, enrollment started on late November/ early December 2020.

- **Descriptive analyses** will be performed on an ongoing basis to gain an understanding of the qualitative and quantitative nature of the data collected and the characteristics of the study participants studied. They will most likely rely on households and participants characteristics, look for potential outliers in the data or a pattern of unlikely data by country and site to help field teams regularly correct data entry issues or improve understanding of study procedures.
- **Intermediate assessment** is to evaluate the efficiency of the 'acute febrile illness' case definition and to assess the recruitment strategy and possibly revise them if necessary. All possible analyses based on data availability and quality will be considered for the intermediate assessment. Regarding primary objective on incidence rate of LASV infection, based on the 6-month follow-up schedule, the analysis will be planned at 4 time points: before and after the first LF season; and before and after the second LF season. Which means there will be one at baseline seropositivity and 3 during seroconversion periods.
- **Interim analysis** will be undertaken at the end of the first full Lassa season to analyse data collected after the first full Lassa season for the primary objectives and all key secondary objectives. A very important assessment during the interim analysis will be that of the baseline seropositivity rates in the different sites and countries. This is critical since detection of high seropositivity rates will lead to countries considering enrolling more subjects or even recruiting patients from different geographic area to avoid incidence under-estimation.
- **Final analysis** would be done around September 2022 – November 2022 at earliest for Nigeria, and September 2023 – November 2023 for the other countries.

4.2 Statistical methodology

Study data were collected and managed using the Research Electronic Data Capture tools (REDCap electronic data capture) hosted at Epicentre[5]. All statistical analyses will be performed on statistical software *R* (R Foundation for Statistical Computing, Vienna, Austria), *version* 4.0.3 (or most recent) using the latest databases extracted from the REDCap server. Most of the data will be summarized overall, by country and by site, and for each cohort.

Quantitative baseline variables will be summarized in a table as number of households, number of participants, number of missing data points, ratio of phone/in-person visits mean and standard deviation (for normally distributed variables) or median and interquartile range-IQR- (for highly skewed data). The summary is to be made for each study cohort (LF disease cohort and LASV infection cohort), overall and stratified by site and country (and even age groups and gender if applicable).
Qualitative variables will be summarized as number of participants and percentage (%).
Sex-age pyramid of confirmed LF cases and infected LF cases will be drawn to highlight the age-sex distribution of the cases and describe any particular pattern using the following age-groups: <5, 5-17, 18-49, 50-79, >79 (years old).
Primary and secondary outcomes expressed in prevalence, frequency of occurrence or case fatality ratio will all be presented as percentages (%) as well with their 95% CIs.

4.2.1. Primary and secondary outcomes

Primary outcomes expressed as incidence rates will be calculated using standard person-time methods with person-time of follow-up as a denominator and 95% confidence intervals calculated using the ***Poisson*** distribution.
Regarding seroprevalence, even though not precised in study protocol as secondary outcome, the adjusted prevalence for laboratory kit error will be calculated if data allows. This is important since it corrects the estimates for test sensitivity and specificity. These two latter measures, along with other critical information on the test kits characteristics (are expected to be) provided by the Bernhard-Nocht-Institut für Tropenmedizin (BNITM) team to allow such an analysis. Those characteristics are summarized in the Table 9. Details on the estimation of the test specificity and sensitivity can be found in the respective kit test evaluations.

	**Serology**	**RT-PCR**
Name of assay	Zalgen	RealStar® Lassa Virus RT-PCR kit v2.0
Positivity threshold	See table on serology interpretation	-

[5] Harris et al., "Research Electronic Data Capture (REDCap)—A Metadata-Driven Methodology and Workflow Process for Providing Translational Research Informatics Support."

Sensitivity (95% CI)	*93.75% [95%CI:89.60-96.31]*	Not found
Specificity (95% CI)	*85.65% [95%CI: 68.25-94.31].*	Not found
Methodology for estimating SE/SP	SE/SP were estimated by FIND in Nigeria and Sierra-Leone. The study used blood samples from known positive and negative Lassa cases. Positive patients were confirmed by RT-PCR in Nigeria and by IgG ELISA in Sierra-Leone).	The analytical specificity with respect to the cross reactivity of the RealStar® Lassa Virus RT-PCR Kit 2.0 was evaluated by testing a panel of genomic RNA/DNA extracted from 17 pathogens with a clinical presentation to that of Lassa virus infection. The analytical specificity with respect to the reactivity of the RealStar® Lassa Virus RT-PCR Kit 2.0 was evaluated by a panel of genomic RNA extracted from different Lassa virus lineages (strains).

Table 9: Serology and RT-PCR tests kit characteristics

Note that the sensitivity and specificity values in the table above are based on the serology kit IGG values only. By the time when this SAP is written, the IgM evaluation was not finalized yet.

The adjusted seroprevalence will then be obtained through the following formula:

$$p_{adj}=\frac{p+\beta-1}{\alpha+\beta-1}$$

With *p* the crude prevalence (without corrections and which is biased if the test kit is not perfect) or more precisely the positive test frequency, β the test specificity and α, its sensitivity.

Regarding the PCR tests, no adjustment is deemed necessary by the time of development of this SAP. The RT-PCR kit 2.0 is known to target both the conserved terminus of the S RNA segment and the downstream glycoprotein precursor (GPC) gene called GPC-assays; and the Large (L) gene. Hence its high sensitivity since GPC-assays were found less sensitive to LASV strains lineages circulating in Benin for instance, while L-assays had a low CT value for this lineage, so were more sensitive, and the other lineages were reported to be detectable almost equally by the two assay types (cf Altona 2.0 manual RealStar Lassa Virus RT-PCR Kit 2.0_WEB_CE_EN-S02.pdf (altona-diagnostics.com)).

Due to sampling methodology in many sites involving cluster sampling, there will be a cluster-effect in the data collected, villages or households representing the clusters. Thus, statistical methods accounting for clustering will be selected depending on the analyses such as Generalized Linear Mixed models (GLMM), known as well as random effect models (or multilevel) and generalized estimating equations (GEE). GLMM models will be preferred depending on the analysis since they work under the assumption of data missing at random (MAR) and given loss to follow-up are highly possible in our

study, we would need models that will still give unbiased results in such cases[6]. The R package GLMMadaptive will be used since it allows having marginal estimates (https://drizopoulos.github.io/GLMMadaptive/).

The risk of symptomatic LF disease or LASV infection (seroconversion) will be examined for several risk factors (age, sex, history of LF disease, occupation, type of housing, contact/sight/consumption of rodents...). Both univariate and multivariate multilevel models will be used to account for clustered data to estimate Incidence rate ratio (IRR).

4.2.2. Exploratory analyses

- Retention rates within site and attack rates within site for each country will be presented as percentages (%) with confidence intervals calculated using *Poisson* regression.
- Logistic regression based on the GLMM approach will be used:
    - to investigate the contribution of the participants/households baseline characteristics deemed relevant besides sex and age-groups (history of LF disease, occupation, type of housing, contact/sight/consumption of rodents...) to the study retention under the definition of retained participants given in 4.2.1 .
    - For the analysis of the risk related to severity where we will look for the best predictors (specific to confirmed cases) of severity such as the symptoms at admission specific to the confirmed cases, the CT values at confirmation…

As for the other multivariate analyses, variables suggested as risk factors in the literature and other variables of interest newly collected in this study, will be included in the multivariate logistic regression analysis.

- Beside the analysis on the severity, it will be important to assess the effect of the evolution of biological measures between admission and discharge (in confirmed cases) on fatal outcomes. Cox regression models (using the *survival package* in R), if assumptions are verified, can be used to address it, otherwise a Poisson regression model will allow to give indication on these biological measures that could be good predictor of death.
- The *EpiEstim package* in R allows detailed outbreak modelling with estimation of the reproduction number as well projection of outbreak dynamics.
- The genomic analyses and phylogenetic tree construction will be made possible with the Beast software. The effect of the LASV strain on the incidence will be assessed if data allow.

4.2.3. Pooling of sites/countries data

[6] Hubbard et al., "To GEE or Not to GEE."

Seven sites were involved in this study across five countries, hence the heterogeneity of the target populations (mainly risk factors) across countries, some of this heterogeneity will be summarized by boxplot per site for a better understanding.

This multicentre study design allows then a greater exposure variability and may improve the generalizability of the results based on the study primary objectives and key secondary objectives. As a result, it will be of a great benefit to pool similar populations to increase power when looking specifically at certain analysis populations such as the confirmed LF cases population where we might have small sample size when looking at each site separately. The data pooling from the different countries will allow in such case to get a total infection rate for the whole hotspot areas in the country (Nigeria) and for the whole endemic-area under study (regional level).

For these analyses, as well as the risk factor analysis for LF disease or infection, as described in the previous paragraph, a weighting of the estimates by site or country, will be necessary to account for the differences between sites/countries in the target population and other underlying characteristics. The analysis is planned in one step, as the data from the different sites/countries is supposed to be standardised at the design stage and will be easily harmonised and pooled.

The multilevel structure of data allowing also within-site/country differences besides the between-site difference, the application of the multilevel models as suggested in the previous paragraphs will account for these two differences to produce the overall estimates. Since the use of random effects models (adjusted for country) or GEE models to analyse pooled data assumes an adequate number of centres (in this case country) set in the literature at a minimum of five (6), we will prefer a site-based rather than country-based pooled analysis, where relevant.

In the case where the one-stage analysis methodology proposed turns out complex to apply because of few events per country, we plan to analyse each country separately and then combining the results using meta-analyses methodology.

4.2.4. Methods for Handling Missing Data

Missing data was decided in protocol not to be imputed and the data to be analysed as they are recorded in the questionnaire/CRF.
However, as a complete data analysis may involve deleting a large proportion of the study data, which may affect the results of the analyses, unless data are missing completely at random (an unrealistic scenario); if more than 10% of the data are missing for one or more key variables, the protocol suggests discussing the impact of the missing data on the analysis and exploring the pattern of missing data.
If there is evidence of bias in the missing data, and variables that are considered good predictors of the missing data are available, the multiple imputation method at the study level may be used through the *mice* package from ***R*** to replace missing values as secondary exploratory analyses. This method performs multiple imputation by the MICE (Multiple Imputation by Chained Equations) procedure

using an iterative multivariate regression technique that replaces missing data with plausible values to estimate more realistic regression coefficients that are not affected by missing values (7). Though originally mainly built for non-clustered data (8), mice version **3.14.0** (mice: Multivariate Imputation by Chained Equations (r-project.org)) now incorporates procedures to handle clustered data and will be used in priority. Based on issues we may encounter; we could explore the *jomo*[7] (https://cran.r-project.org/web/packages/jomo/jomo.pdf) or *jointai* [8](https://arxiv.org/abs/1907.10867) packages that both work under joint modelling framework allowing handling of missing data in multilevel models.

If the multiple imputation method is used, a sensitivity analysis will be carried out comparing results from the complete case analysis (where records with missing data will be dropped) and the full set analysis (with imputed data).

In addition, depending on the analysis, missing information may be treated differently or not considered at all. For instance:

- In the incidence rate of LF disease calculation, given the denominator is the total time the population was at risk of and being watched for disease, we will deliberately discard all data following a temporary termination as we will not be able to assess if the person experienced Lassa fever while outside of the study. This should prevent overestimation of the person-years and underestimation of the incidence rate (cf Primary outcomes). However, if the person experience Lassa fever after resuming the study, s/he will be included in both the numerator and denominator of the incidence rate.
- In the case of regression analyses, missing data handling will depend on the type of regression chosen. In the GEE regression it is assumed data are missing completely at random i.e., the probability an observation is missing is independent of any other observation. While in the random effect models the assumption is that data are missing at random i.e., the probability that an observation is missing may depend on values at other times or on the values of other variables in the dataset.

	**Missing data handling**	**Methodology**
Generic calculation	Omit if <10% missing	List-wise deletion
Generic calculation	Imputation	Multiple imputation

[7] Quartagno, Grund, and Carpenter, "Jomo."
[8] "Erler: JointAI: Joint Analysis and Imputation of... - Google Scholar."

Incidence rate for confirmed LF	If a participant has moved away or was lost of follow-up, even if they come back, we should consider their data until the last time point, they were seen, unless they experience Lassa fever after their come back.	Right-censoring at the last point participant was seen before moving or being lost of follow-up for a sufficient time to get infected then cured.
Random effect models (mixed)	Considered as missing at random.	
GEE	Considered as missing completely at random (stronger assumption and unrealistic scenario)	No imputation: drops missing points of a participant; not all points if any missing

Table 10: Different scenarios for missing data handling

4.2.5. Confounding factors

A few potential confounders are to be considered for the aforementioned analyses:

- Age: analyses on seroconversion mainly could be highly confounded by age since adults are more likely to get immunity though the years of exposure to Lassa fever virus.
- Sex: except for countries or sites where alternative methods were applied to ensure inclusion of entire households, it is often seen in such studies a higher proportion of female participants that can be interpreted with the absence of household male members during the inclusion visit for instance. There may be differential recruitment by age as well as for sex, with young and working aged people more likely to be out of the home, or differential LTFU if young people are away for long periods.
- Month/season of follow-up: as stated above, there could be an overlap with the high LASV transmission period if our recruitment was to be delayed. We should then take this into account since it could lead to overestimation of the baseline seroprevalence and under-estimation of infection and symptomatic incidence. Plus, the seasonality of the LV transmission in the localities of interest and the variation in the follow-up activities during the study period, could also lead some more biases. A way to consider these biases could be to make a time-varying curve for the incidence. But this could be difficult if we only have a few number of cases. This will then be explored if data allows.
- Comorbidities: for confirmed cases referred to hospital, CRF N.5 (Confirmed case assessment) will be filled with information on the patient's underlying comorbidities such as pregnancy, diabetes, or chronic renal/liver disease collected. The analyses on severity and fatal outcome will include the presence of one or multiple comorbidities to assess any possible confounding effect.
- Site/country: regarding estimation of the overall incidence rates, pooling of the data from the different sites and countries will be necessary, hence the weighting of the estimates by site or country to get at a total incidence rate.

5. Preparation of tables, listings and figures

The intended list of tables and figures that will be produced following the Strengthening the Reporting of Observational studies in Epidemiology (STROBE) guidelines will be presented in another document as appendix to this SAP (Appendix 2). Despite the (non-exhaustive) enumeration of these outputs at the stage of the writing of the SAP, it may be necessary to change the table contents after analysis, as appropriate, in the light of the data available and the amendment to the planned statistical analyses.

An overview table will be produced summarizing the different studies per country/site and per cohort alongside the number of participants recruited, the dates of enrolment start and last visit, the mean duration of follow-up. This will allow having an idea of the differences in the different studies and the level of standardization needed estimating the overall incidence.
A flowchart showing the disposition of study participants, will be then presented. This follow-up diagram will show the number of participants eligible, enrolled, those who attended first follow-up visit, and will show participants who terminated study earlier, for each cohort. Participants who became LF confirmed cases or LASV infected (according to the cohort) will also be presented down the diagram as well as the number of participants considered as retained. The flowchart is to be presented overall and for each country.
The first characteristics of interest will involve households. Tables will summarize HH characteristics including the mean number of members, the type of housing, the presence of a healthcare worker in the HH, the presence/sight and consumption of rodents within the HH.
Then, participants demographics and baseline characteristics, including age, sex, occupation and historic of LF disease or contact with rodents, will be described for each cohort.
An age-sex pyramid will highlight the age-sex distribution among participants overall and then within each country/site.
Regarding the primary outcomes and key secondary outcomes, the overall and per-country incidence rates per 1000 person-years will be presented in tables and potentially on maps.
Majority of the secondary outcomes and other exploratory analyses results will be summarized in tables, expect for the monthly incidence of confirmed LF cases that will be plotted overall then by site.

Besides tables and figures, several critical information will be available in listings. Lists of HH members who were approached but ended up not being eligible, those who early discontinued the study follow-up, and the reasons of discontinuation will be provided, those who died during follow-up and the deaths causes, those who were not retained (as per definition). Regarding confirmed cases, SHNL cases, pregnant women and paediatric cases will also be listed.
In addition to listing of participants, major and minor protocol deviations will be also presented in lists.

6 Bibliography

"Erler: JointAI: Joint Analysis and Imputation of... - Google Scholar." Accessed April 27, 2022. https://scholar.google.com/scholar_lookup?arxiv_id=1907.10867.

Harris, Paul A., Robert Taylor, Robert Thielke, Jonathon Payne, Nathaniel Gonzalez, and Jose G. Conde. "Research Electronic Data Capture (REDCap)—A Metadata-Driven Methodology and Workflow Process for Providing Translational Research Informatics Support." *Journal of Biomedical Informatics* 42, no. 2 (April 1, 2009): 377–81. https://doi.org/10.1016/j.jbi.2008.08.010.

Hubbard, Alan E., Jennifer Ahern, Nancy L. Fleischer, Mark Van der Laan, Sheri A. Satariano, Nicholas Jewell, Tim Bruckner, and William A. Satariano. "To GEE or Not to GEE: Comparing Population Average and Mixed Models for Estimating the Associations Between Neighborhood Risk Factors and Health." *Epidemiology* 21, no. 4 (2010): 467–74.

Miller, Ann C., Peter Rohloff, Alexandre Blake, Eloin Dhaenens, Leah Shaw, Eva Tuiz, Francesco Grandesso, Carlos Mendoza Montano, and Dana R. Thomson. "Feasibility of Satellite Image and GIS Sampling for Population Representative Surveys: A Case Study from Rural Guatemala." *International Journal of Health Geographics* 19, no. 1 (December 5, 2020): 56. https://doi.org/10.1186/s12942-020-00250-0.

Okokhere, Peter, Andres Colubri, Chukwuemeka Azubike, Christopher Iruolagbe, Omoregie Osazuwa, Shervin Tabrizi, Elizabeth Chin, et al. "Clinical and Laboratory Predictors of Lassa Fever Outcome in a Dedicated Treatment Facility in Nigeria: A Retrospective, Observational Cohort Study." *The Lancet Infectious Diseases* 18, no. 6 (June 1, 2018): 684–95. https://doi.org/10.1016/S1473-3099(18)30121-X.

Quartagno, Matteo, Simon Grund, and James Carpenter. "Jomo: A Flexible Package for Two-Level Joint Modelling Multiple Imputation." *The R Journal* 11, no. 2 (2019): 205–28.

Richmond, J Kay, and Deborah J Baglole. "Lassa Fever: Epidemiology, Clinical Features, and Social Consequences." *BMJ : British Medical Journal* 327, no. 7426 (November 29, 2003): 1271–75.

TURNER, ANTHONY G, ROBERT J MAGNANI, and MUHAMMAD SHUAIB. "A Not Quite as Quick but Much Cleaner Alternative to the Expanded Programme on Immunization (EPI) Cluster Survey Design." *International Journal of Epidemiology* 25, no. 1 (February 1, 1996): 198–203. https://doi.org/10.1093/ije/25.1.198.

7 Appendices

SAP-Appendix 1: details on sampling methodology

SAP-Appendix 2: Tables, figures and listings

Country	Sites (districts)	Cohort(s)	Number of expected participants (Number of clusters)	Sampling method	Enrolment start
Guinea	Faranah	Infection	1,000 (32)	Purposively sampling of clusters + Number of households proportional to cluster size + systematic sampling within cluster	June 2021
Benin	1 site in Nothern Benin with 3 districts (Tchaourou, Parakou, Natitingou)	Disease and Infection	5,000 (80)	Two-stage random sampling of 80 villages among 103 listed villages + GPS sampling within cluster	July 2021
Liberia	Bong County	Disease and Infection	5,000 (3)	Purposively sampling of clusters + Number of households proportional to cluster size + GPS sampling within cluster	May 2021
Nigeria	3 sites:<br>Irrua (Edo state)<br>Abakaliki (Ebonyi state)<br>Owo (Ondo state)	Disease (Irrua site only) and Infection (all 3 sites)	7,000 (27)	Purposively sampling of clusters + Number of households proportional to cluster size + systematic sampling within cluster	December 2020
Sierra Leonne	4 districts surrounding KGH	Disease and Infection	5000 (18)	2-stage cluster sampling of clusters (1st stage: Section stratified by district, 2nd stage: village) + exhaustive sampling within cluster	June 2021

Table 7-1:Study schema, sites and their specificity

SAP ENABLE LASSA STUDY

Final Audit Report 2022-09-19

Created:	2022-08-18
By:	Marine Durthaler (marine.durthaler@epicentre.msf.org)
Status:	Signed
Transaction ID:	CBJCHBCAABAAWZBtwP_7dXdOhLf-3BLoOejid_ydZAA4

"SAP ENABLE LASSA STUDY" History

Document created by Marine Durthaler (marine.durthaler@epicentre.msf.org)
2022-08-18 - 9:00:29 AM GMT- IP address: 93.29.240.150

Document emailed to Anton CAMACHO (anton.camacho@epicentre.msf.org) for signature
2022-08-18 - 9:05:45 AM GMT

Document emailed to Robert NSAIBIRNI (robert.nsaibirni@epicentre.msf.org) for signature
2022-08-18 - 9:05:45 AM GMT

Document emailed to adebola.olayinka@ncdc.gov.ng for signature
2022-08-18 - 9:05:45 AM GMT

Document emailed to david_wohl@med.unc.edu for signature
2022-08-18 - 9:05:45 AM GMT

Document emailed to ayola-akim.adegnika@uni-tuebingen.de for signature
2022-08-18 - 9:05:45 AM GMT

Document emailed to donkumfel@gmail.com for signature
2022-08-18 - 9:05:46 AM GMT

Document emailed to cmagassouba01@gmail.com for signature
2022-08-18 - 9:05:46 AM GMT

Document emailed to suzanne.penfold@p-95.com for signature
2022-08-18 - 9:05:46 AM GMT

Document emailed to mywilliams@mmarcro.org for signature
2022-08-18 - 9:05:46 AM GMT

Document emailed to guenther@bnitm.de for signature
2022-08-18 - 9:05:46 AM GMT

Adobe Acrobat Sign

Document emailed to Gabrielle Breugelmans (gabrielle.breugelmans@cepi.net) for signature
2022-08-18 - 9:05:46 AM GMT

Email viewed by cmagassouba01@gmail.com
2022-08-18 - 9:05:57 AM GMT- IP address: 66.249.93.16

Email viewed by Robert NSAIBIRNI (robert.nsaibirni@epicentre.msf.org)
2022-08-18 - 9:06:37 AM GMT- IP address: 154.72.206.59

Email viewed by david_wohl@med.unc.edu
2022-08-18 - 9:10:29 AM GMT- IP address: 104.28.39.149

Document e-signed by Robert NSAIBIRNI (robert.nsaibirni@epicentre.msf.org)
Signature Date: 2022-08-18 - 9:14:29 AM GMT - Time Source: server- IP address: 154.72.206.59

Email viewed by ayola-akim.adegnika@uni-tuebingen.de
2022-08-18 - 9:46:44 AM GMT- IP address: 66.249.93.16

Signer ayola-akim.adegnika@uni-tuebingen.de entered name at signing as aa.adegnika
2022-08-18 - 10:07:11 AM GMT- IP address: 41.138.89.197

Document e-signed by aa.adegnika (ayola-akim.adegnika@uni-tuebingen.de)
Signature Date: 2022-08-18 - 10:07:12 AM GMT - Time Source: server- IP address: 41.138.89.197

Signer david_wohl@med.unc.edu entered name at signing as David Alain Wohl
2022-08-18 - 1:24:26 PM GMT- IP address: 162.198.202.210

Document e-signed by David Alain Wohl (david_wohl@med.unc.edu)
Signature Date: 2022-08-18 - 1:24:28 PM GMT - Time Source: server- IP address: 162.198.202.210

Email viewed by Gabrielle Breugelmans (gabrielle.breugelmans@cepi.net)
2022-08-18 - 7:28:04 PM GMT- IP address: 89.8.65.151

Email viewed by Anton CAMACHO (anton.camacho@epicentre.msf.org)
2022-08-20 - 8:53:53 AM GMT- IP address: 104.28.34.167

Email viewed by Gabrielle Breugelmans (gabrielle.breugelmans@cepi.net)
2022-08-21 - 9:11:00 AM GMT- IP address: 104.28.30.78

Email viewed by mywilliams@mmarcro.org
2022-08-21 - 9:27:48 AM GMT- IP address: 81.150.176.123

Signer mywilliams@mmarcro.org entered name at signing as Margaret Williams
2022-08-21 - 9:28:56 AM GMT- IP address: 81.150.176.123

Document e-signed by Margaret Williams (mywilliams@mmarcro.org)
Signature Date: 2022-08-21 - 9:28:57 AM GMT - Time Source: server- IP address: 81.150.176.123

Email viewed by cmagassouba01@gmail.com
2022-08-21 - 6:17:00 PM GMT- IP address: 66.249.93.10

Email viewed by suzanne.penfold@p-95.com
2022-08-22 - 7:20:12 AM GMT- IP address: 46.233.83.184

Signer suzanne.penfold@p-95.com entered name at signing as S. C. Penfold
2022-08-23 - 6:18:41 AM GMT- IP address: 85.135.208.82

Document e-signed by S. C. Penfold (suzanne.penfold@p-95.com)
Signature Date: 2022-08-23 - 6:18:43 AM GMT - Time Source: server- IP address: 85.135.208.82

Email viewed by Anton CAMACHO (anton.camacho@epicentre.msf.org)
2022-08-23 - 8:31:34 AM GMT- IP address: 104.28.34.167

Email viewed by adebola.olayinka@ncdc.gov.ng
2022-08-23 - 2:37:17 PM GMT- IP address: 197.210.205.26

Signer adebola.olayinka@ncdc.gov.ng entered name at signing as Adebola Olayinka
2022-08-23 - 2:40:22 PM GMT- IP address: 197.210.205.26

Document e-signed by Adebola Olayinka (adebola.olayinka@ncdc.gov.ng)
Signature Date: 2022-08-23 - 2:40:24 PM GMT - Time Source: server- IP address: 197.210.205.26

Email viewed by Gabrielle Breugelmans (gabrielle.breugelmans@cepi.net)
2022-08-24 - 9:19:17 AM GMT- IP address: 104.28.31.34

Email viewed by cmagassouba01@gmail.com
2022-08-24 - 10:01:15 AM GMT- IP address: 66.249.93.184

Email viewed by Anton CAMACHO (anton.camacho@epicentre.msf.org)
2022-08-24 - 2:14:55 PM GMT- IP address: 104.28.34.164

Signer cmagassouba01@gmail.com entered name at signing as Prof Magassouba N'faly
2022-08-26 - 4:41:17 PM GMT- IP address: 197.149.243.121

Document e-signed by Prof Magassouba N'faly (cmagassouba01@gmail.com)
Signature Date: 2022-08-26 - 4:41:18 PM GMT - Time Source: server- IP address: 197.149.243.121

Email viewed by Anton CAMACHO (anton.camacho@epicentre.msf.org)
2022-08-27 - 2:38:21 PM GMT- IP address: 104.28.34.155

Document e-signed by Anton CAMACHO (anton.camacho@epicentre.msf.org)
Signature Date: 2022-08-27 - 7:19:21 PM GMT - Time Source: server- IP address: 45.149.228.110

Email viewed by Gabrielle Breugelmans (gabrielle.breugelmans@cepi.net)
2022-08-27 - 9:19:23 PM GMT- IP address: 62.199.211.12

Adobe Acrobat Sign

Email viewed by Gabrielle Breugelmans (gabrielle.breugelmans@cepi.net)
2022-08-30 - 10:08:38 AM GMT- IP address: 188.95.246.159

Document e-signed by Gabrielle Breugelmans (gabrielle.breugelmans@cepi.net)
Signature Date: 2022-08-30 - 10:09:33 AM GMT - Time Source: server- IP address: 188.95.246.159

Email viewed by guenther@bnitm.de
2022-08-30 - 11:49:33 AM GMT- IP address: 82.207.187.7

Signer guenther@bnitm.de entered name at signing as Stephan Günther
2022-08-30 - 11:51:41 AM GMT- IP address: 82.207.187.7

Document e-signed by Stephan Günther (guenther@bnitm.de)
Signature Date: 2022-08-30 - 11:51:43 AM GMT - Time Source: server- IP address: 82.207.187.7

Email viewed by donkumfel@gmail.com
2022-09-05 - 11:09:48 AM GMT- IP address: 66.249.93.16

Email viewed by donkumfel@gmail.com
2022-09-19 - 6:48:40 PM GMT- IP address: 66.249.93.176

Signer donkumfel@gmail.com entered name at signing as Donald S. Grant
2022-09-19 - 6:50:11 PM GMT- IP address: 197.215.23.175

Document e-signed by Donald S. Grant (donkumfel@gmail.com)
Signature Date: 2022-09-19 - 6:50:12 PM GMT - Time Source: server- IP address: 197.215.23.175

Agreement completed.
2022-09-19 - 6:50:12 PM GMT

Adobe Acrobat Sign
